# Targeting the ANGPTL4/NRP1/ABL1/RAD51 axis reverses cisplatin resistance by impairing DNA damage repair in head and neck cancer

**DOI:** 10.1073/pnas.2510265123

**Published:** 2026-03-26

**Authors:** Emmanuel B. Asiedu, Ajay Kumar, Alexander Choi, Derek Osorio Luciano, Kevin Lo, Deepti Sharma, Tao Ma, Feyruz Rassool, Akrit Sodhi, Silvia Montaner

**Affiliations:** ^a^Department of Oncology and Diagnostic Sciences, School of Dentistry, University of Maryland, Baltimore, MD 21201; ^b^The Wilmer Eye Institute, Johns Hopkins University School of Medicine, Baltimore, MD 21287; ^c^Department of Radiation Oncology, School of Medicine, University of Maryland, Baltimore, MD 21201; ^d^Greenebaum Comprehensive Cancer Center, University of Maryland, Baltimore, MD 21201

**Keywords:** angiopoietin-like 4, squamous cell carcinoma of head and neck, cisplatin, RAD51 recombinase, drug resistance

## Abstract

Drug chemoresistance remains a major reason of treatment failure in cancer patients. In head and neck squamous cell carcinoma (HNSCC), the seventh most common cancer worldwide, cisplatin chemotherapy remains the gold standard for advanced tumors but often faces loss of responsiveness and the drawback of relapse. We previously showed that the metabolic and angiogenic factor angiopoietin-like 4 (ANGPTL4) is a molecular biomarker of oral dysplasia and HNSCC. We also found that through interaction with Neuropilin 1 (NRP1), ANGPTL4 activates proliferative and migratory pathways that contribute to HNSCC development. Using HNSCC xenografts, patient tumor-derived organoids, tumor spheroids, and HNSCC cell lines, CAL27, HN13, and HN4, here we provide evidence of the role of ANGPTL4 in the development of platinum-based chemoresistance in HNSCC through the promotion of DNA damage response (DDR) and homologous recombination (HR). ANGPTL4 enhanced these mechanisms by promoting phosphorylation of RAD51 recombinase in Tyr^315/54^ through an NRP1/ABL1-dependent mechanism. Pharmacologic inhibition of NRP1 or ABL1 reversed ANGPTL4-mediated DDR and HR, and increased HNSCC cell death in combination with cisplatin, in vitro and in vivo. Our results reveal a role for ANGPTL4 in RAD51-dependent DNA repair and suggest that ANGPTL4/NRP1/ABL1/RAD51 may serve as an alternative therapeutic target for HNSCC.

## Significance

Here, we identify a molecular pathway that drives cisplatin resistance in head and neck squamous cell carcinoma (HNSCC). The angiogenic factor angiopoietin-like 4 (ANGPTL4) engages Neuropilin1 to activate ABL1, which in turn phosphorylates and activates the DNA recombinase and homologous recombination protein, RAD51. This enables HNSCC cells to survive cisplatin-induced genotoxic stress. These results mechanistically demonstrate a role for ANGPTL4 and a cellular signaling axis to overcome chemoresistance in HNSCC. These findings also have potential implications for other ANGPTL4-overexpressing tumors.

Overcoming drug chemoresistance is a serious challenge encountered during cancer patient treatment and one of the major tasks of individualized cancer medicine (1, 2). In many neoplasias, including those with robust initial responses, cancer cells eventually acquire the capacity to evade drug cytotoxicity, compromising patient survival. Substantial progress has been accomplished on the elucidation of the genetic and biochemical mechanisms hijacked by cancer cells to facilitate drug tolerance. However, due to the diverse nature of the intrinsic and acquired molecular changes that lead to drug insensitivity and the genetic heterogeneity of patients and tumor cell populations, advances in the field are still insufficient and cancer chemoresistance remains an obstacle for therapy success.

The use of patient-derived tumor cell organoids and high-throughput tissue analysis are changing the landscape of the investigations on tumor biomarkers and treatment design, including the ones related to chemoresistance (3). Culprit gene products unsettling drug tolerance are involved in events including drug influx or efflux, drug inactivation, apoptosis, autophagy, DNA repair, epithelial–mesenchymal transition, and cancer stemness. These drug desensitizing proteins are exploited by cancer cells to sustain growth and dissemination; therefore, the effective inhibition of their activity is crucial to improve treatment efficacy and clinical outcomes (4).

In this regard, we have previously reported that the metabolic, proangiogenic, and protumorigenic protein, angiopoietin-like 4 (ANGPTL4), plays an important role in cancer development and progression (5–9). ANGPTL4 belongs to the family of human angiopoietin-like (ANGPTL) proteins, which are associated with cell energy metabolism, inflammation, stem cell maintenance, vascular homeostasis, and cancer (10, 11). Once secreted, ANGPTL4 is susceptible to cleavage into two major regions, a coil-coiled N-terminal domain and a fibrinogen-like C-terminal domain, with different functionalities (12). Many studies on ANGPTL4 are related to its activity as an adipokine and putative therapeutic target for cardiovascular and metabolic disorders (11, 13). Indeed, ANGPTL4 has been shown to inhibit lipoprotein lipase and to be a master regulator of circulating TGs levels and body TGs partitioning (14). It also controls glucose metabolism and circulating high-density lipoprotein (HDL) cholesterol levels (15). Recent reports show that ANGPTL4 participates in inflammatory diseases such as lung injury, heart disease, gastrointestinal disease, and pancreatitis. However, the specific relationship between this ligand and inflammation is still not well understood (16).

In addition, ANGPTL4 is part of the HIF-1 transcriptional response elicited in tissues to adapt to low oxygen conditions (7, 17). Our group and others demonstrated that HIF-mediated ANGPTL4 upregulation is critical in angiogenesis and vascular leakage, playing an important role in ocular neovascularization, edema, wound healing, and tumor growth (5–9, 18–24). In cancer, ANGPTL4 is a biomarker of poor prognosis in many tumors, including breast cancer, ovarian cancer, colon cancer, gastric cancer, prostate cancer, and head and neck squamous cell carcinoma (HNSCC) (25). ANGPTL4 induces multiple protumorigenic mechanisms including tumor cell proliferation, tumor cell migration, altered redox balance, and metastasis. Some of those processes are mediated through the binding of ANGPTL4 to neuropilin 1/2 (NRP1/2) or α5β1 integrin, leading to the activation of autocrine and paracrine signaling pathways in tumor cells and stroma cells, including the vascular endothelium (21, 26).

One of the adverse effects that HIF-1 activation mediates in tumor behavior is the development of anticancer drug resistance (27). In the case of cisplatin, a first-line chemotherapeutic drug, HIF-1 impairs its anticancer effects by upregulating genes and intracellular networks that alter drug efflux or detoxification, DNA damage repair, and apoptosis (28). Interestingly, many cancer types that eventually fail cisplatin treatment have upregulated ANGPTL4 expression as part of their proteomic signature (29). In particular, ANGPTL4 has been found to be a molecular marker associated with poor clinical prognosis in HNSCC, one of the most common cancers worldwide (30–36). HNSCC comprises an aggressive group of tumors arising from the mucosal lining of the oral cavity, pharynx, larynx, nasal cavity, and salivary glands (37–39). In the United States, around 54,000 new cases of HNSCC are diagnosed every year and around 65 to 70% of the diagnosis occur with the presence of locally advanced (stage III) and distantly metastatic disease (stage IV). In these situations, treatment usually includes surgical intervention and/or cisplatin-based concurrent chemoradiotherapy, which often faces the onset of chemoresistance. Today, patients with advanced HNSCC demonstrate a 5-y overall survival of 10 to 50%, with individuals with recurrence or distal metastases showing the worse prognosis (40). Developing more effective therapeutic approaches is crucial to overcome these adverse clinical outcomes and improve patient survival.

Here, we use HNSCC as a model for upregulated ANGPTL4 expression to investigate whether ANGPTL4 affects cisplatin resistance, the molecular mechanism involved, and its role as a putative biomarker to sensitize cancer cells to cisplatin therapy.

## Results

### ANGPTL4 Decreases Sensitivity to Cisplatin in HNSCC.

**Fig. 1.**
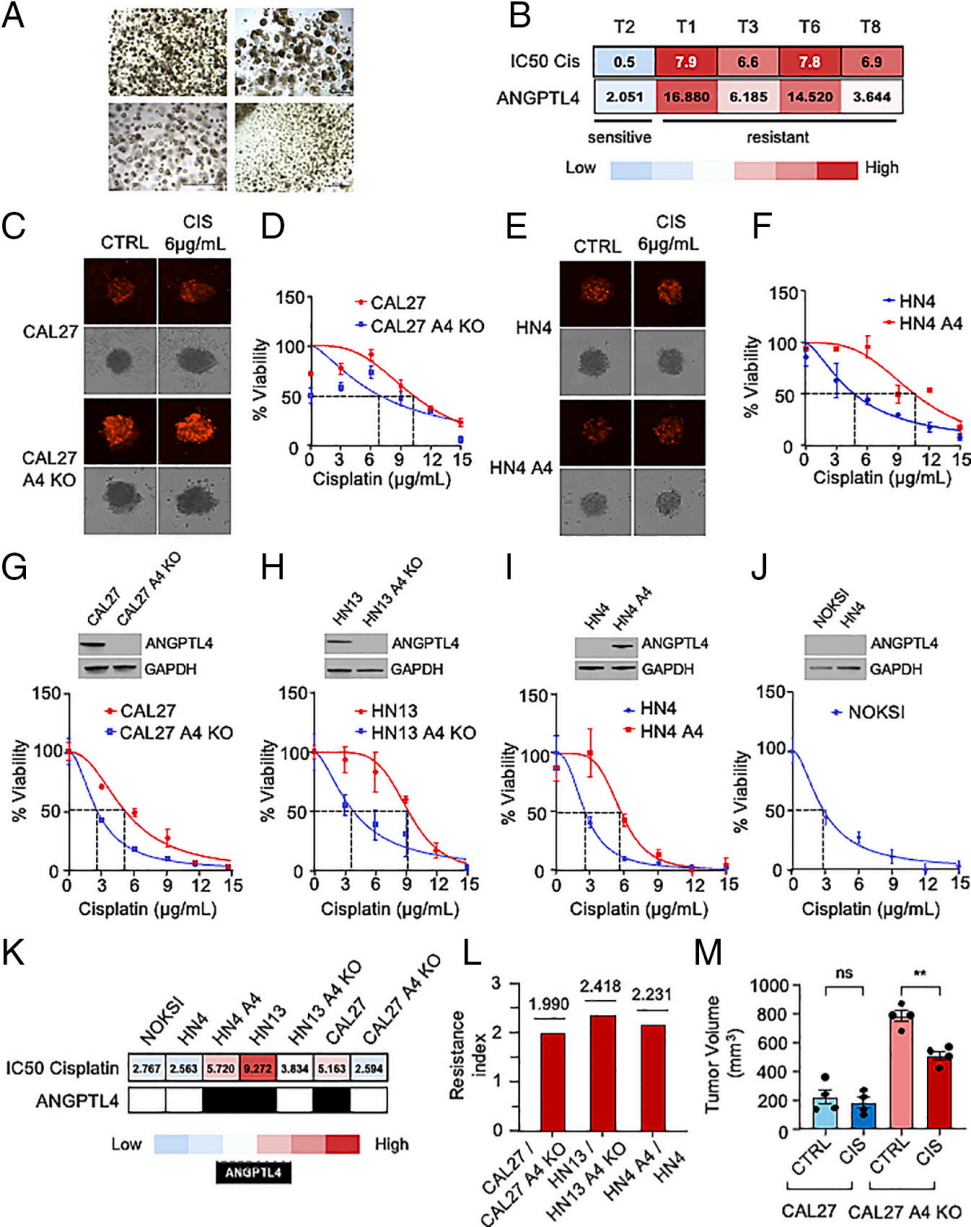
Correlation between ANGPTL4 expression and decreased cisplatin sensitivity and cell apoptosis in HNSCC models. (*A*) Representative brightfield micrographs of HNSCC PTDOs, obtained by biopsy or resection in ref. 41. (Scale bar, 500 μm.) (*B*) Heatmap showing IC50 values for cisplatin of HNSCC PTDOs and their ANGPTL4 mRNA expression. (*C* and *D*) Brightfield and fluorescent micrographs of CAL27 and CAL27 A4 KO cell–derived spheroids treated with (6 µg/mL) cisplatin and stained with propidium iodide, PI (*C*), and determination of the IC50 using Cell Titer Glo assay using (0, 3, 6, 9, 12, and 15 µg/mL) cisplatin (*D*). (*E* and *F*) Brightfield and fluorescent micrographs of HN4 and HN4 A4 cell–derived spheroids treated with (6 µg/mL) cisplatin and stained with PI (*E*), and determination of the IC50 using (0, 3, 6, 9, 12, and 15 µg/mL) cisplatin (*F*). (*G*–*J*) Determination of the cisplatin IC50 in CAL27 and CAL27 A4 KO (*G*), HN13 and HN13 A4 KO (*H*), HN4 and HN4 A4 (*I*), or NOKSI (*J*) treated with cisplatin (0, 3, 6, 9, 12, and 15 µg/mL). Immunoblots show ANGPTL4 expression in cell lines. (*K*) Heat map showing IC50 values for cisplatin from panels (*G*–*J*). Red: high IC50, blue: low IC50. Black: ANGPTL4 overexpression, white: ANGPTL4-null expression. (*L*) Resistance indices for CAL27/CAL27 A4 KO, HN13/HN13 A4 KO, and HN4 A4/HN4 calculated from IC50 for cisplatin from panels (*G*–*I*). (*M*) CAL27 and CAL27 A4 KO cisplatin response in vivo. Nude mice were injected subcutaneously in flanks with 2 × 10^6^ cells and treated with cisplatin 5 mg/kg i.p. (n = 5). Tumor volumes were calculated as described in *Methods*. Data are presented as mean ± SEM. ns *P* > 0.05, *P < 0.05, **P < 0.01, ***P < 0.001, and ****P < 0.0001.

To identify mechanisms underlying HIF-1-dependent cisplatin chemoresistance in HNSCC, we used a panel of HNSCC patient tumor-derived organoids (PTDOs) previously described, genomically characterized, and screened for sensitivity to cisplatin and other cancer drugs (41, 42). Organoids derived from head and neck locations including the tongue, larynx, oral cavity, or gingiva were obtained from HPV-negative, treatment-naive HNSCC patients and cultured in vitro (Fig. 1*A*) (41). Interestingly, examination of the RNA profile of PTDOs T1, T2, T3, T6, and T8, analyzed by RNA-sequencing (CEL-Seq), and their cisplatin half-maximal inhibitory concentration (IC_50_) showed a correlation between the levels of expression of the HIF-1 effector and HNSCC biomarker, ANGPTL4, and their sensitivity to the drug (Fig. 1*B*) (41).

Our group had reported that ANGPTL4 is elevated both in oral dysplasia and HNSCC and is involved in HNSCC cell proliferation and migration (8, 9). We then set out to assess if ANGPTL4 could also play a role in cisplatin chemoresistance in this cancer. To do this, we used three HNSCC cell lines: CAL27 and HN13, derived from tongue HNSCCs (T4N1M0 and T2N2M0, respectively) and overexpressing ANGPTL4, and HN4, also derived from tongue HNSCC (T4N1M0), but not showing detectable ANGPTL4 expression (8, 9). We generated three-dimensional (3D) in vitro tumor spheroids using CAL27 and its respective CRISPR-engineered ANGPTL4 knock-out cell line (CAL27 A4 KO) as well as HN4 and the same cell line ectopically overexpressing ANGPTL4 (HN4 A4). We exposed these 3D tumor spheroids to cisplatin and evaluated tumor cell viability. Interestingly, downregulation of ANGPTL4 expression in CAL27 A4 KO led to an increase in cisplatin sensitivity of the 3D cellular structures compared to the parental CAL27 (Fig. 1 *C* and *D*). Conversely, overexpression of ANGPTL4 in HN4 A4 led to improved cell viability and increased the IC_50_ when cells were exposed to cisplatin compared to the parental HN4 (Fig. 1 *E* and *F*). Standard cultures of CAL27 A4 KO, HN13 A4 KO (also obtained through CRISPR-mediated knock-out of *ANGPTL4*), and HN4 A4 showed similar results for cell viability and drug sensitivity when treated with cisplatin, compared to their respective parental controls (Fig. 1 *G*–*I*). Interestingly, sensitivity to cisplatin in the cell lines with no ANGPTL4 expression (i.e., CAL27 A4 KO, HN13 A4 KO, and HN4) was similar to that of spontaneously immortalized normal human oral keratinocytes, NOKSI, which do not express ANGPTL4 (Fig. 1*J*) (43). A heat map including the IC_50_ values for cisplatin of all these cell lines and their levels of ANGPTL4 expression is shown in Fig. 1*K*. Their resistance index $\left(\text{Resistance}\;\text{index}\;(\text{RI})=\frac{IC50\;\mathrm{of}\;\mathrm{resistant}\;\mathrm{cell}}{IC50\;\mathrm{of}\;\mathrm{sensitive}\;\mathrm{cell}}\right)$ is presented in Fig. 1*L*, showing that CAL27, HN13, and HN4 A4 are about twofold more resistant to cisplatin compared to their ANGPTL4-null counterparts. The same sensitivity profile to cisplatin was manifested when CAL27 and CAL27 A4 KO were injected into the flanks of immunocompromised mice and tumors were generated. Fig. 1*M* shows that CAL27 A4 KO xenografts showed much higher sensitivity to (5 mg/kg) cisplatin than CAL27 tumors, by the end of treatment. Collectively, these findings demonstrate that elevated ANGPTL4 expression correlates with decreased tumor cell apoptosis and decreased cisplatin chemosensitivity in both in vitro and in vivo models of HNSCC tumorigenesis.

### ANGPTL4 Increases DNA Damage Response and Homologous Recombination Repair upon Cisplatin Treatment in HNSCC.

**Fig. 2.**
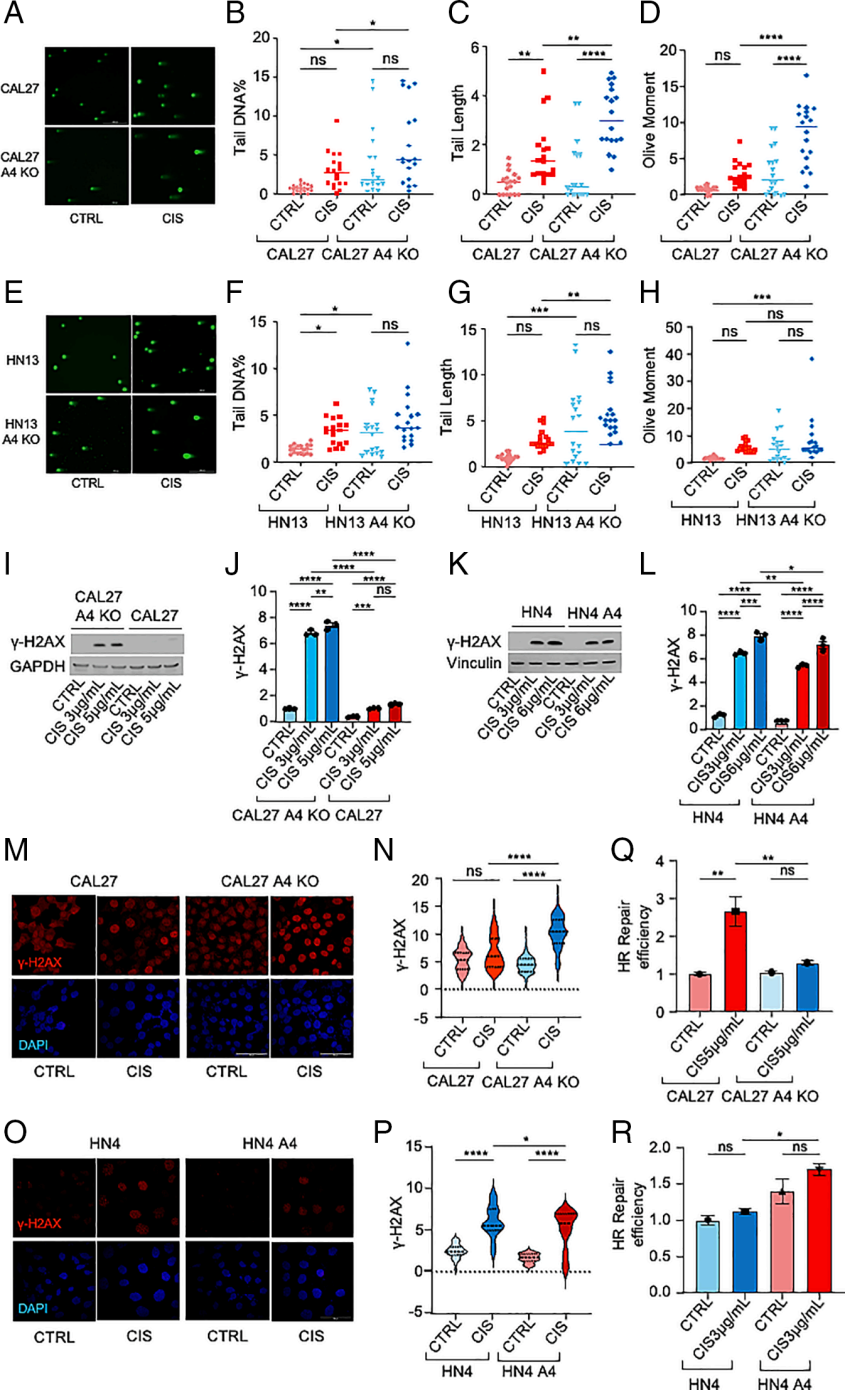
ANGPTL4 increases DNA damage response and homologous recombination repair in HNSCC cells treated with cisplatin. (*A*–*D*) Representative pictures of comets (*A*) from neutral comet assay in CAL27 (red) and CAL27 A4 KO (blue) cells treated with 3 µg/mL cisplatin for 48 h. Comet parameters: tail length (*B*), tail DNA percentage (*C*), and olive moment (*D*) are provided. (Scale bar, 200 µm.) DNA stained with SYBR green. (*E*–*H*) Representative pictures of comets (*E*) from neutral comet assay in HN13 (red) and HN13 A4 KO (blue) cells treated with (5 µg/mL) cisplatin for 48 h. Comet parameters: tail length (*F*), tail DNA percentage (*G*), and olive moment (*H*) are provided. (Scale bar, 200 µm.) DNA stained with SYBR green. (*I* and *J*) Western blot (*I*) and densitometric (*J*) analysis of DNA damage marker γ-H2AX in CAL27 (red) and CAL27 A4 KO (blue) cells following 24 h treatment with (3 µg/mL and 5 µg/mL) cisplatin. (*K* and *L*) Western blot (*K*) and densitometric (*L*) analysis of γ-H2AX in HN4 (blue) and HN4 A4 (red) cells following 24 h treatment with (3 µg/mL and 6 µg/mL) cisplatin. (*M*) Immunofluorescence staining for γ-H2AX (red) in CAL27 and CAL27 A4 KO following 24 h treatment with 5 µg/mL cisplatin. (Scale bar, 15 µm.) Nuclei stained with DAPI. (*N*) Quantification of immunofluorescence intensity from at least 40 cells in (*M*). (*O*) Immunofluorescence staining for γ-H2AX (red) in HN4 and HN4 A4 following 24 h treatment with 5 µg/mL cisplatin. (Scale bar, 50 µm.) Nuclei stained with DAPI. (*P*) Quantification of immunofluorescence intensity from at least 40 cells in (*O*). (*Q*) Relative efficiency of extrachromosomal homologous recombination repair in CAL27 (red) and CAL27 A4 KO (blue) cells treated with (3 µg/mL and 5 µg/mL) cisplatin. (*R*) Relative efficiency of extrachromosomal homologous recombination repair in HN4 (blue) and HN4 A4 (red) cells treated with (3 µg/mL and 6 µg/mL) cisplatin. Data are presented as mean ± SEM. ns *P* > 0.05, *P < 0.05, **P < 0.01, ***P < 0.001, and ****P < 0.0001.

We next set out to determine how ANGPTL4 influences cisplatin chemoresistance in HNSCC. We first examined whether ANGPTL4 expression affected the uptake or export of this drug in HNSCC (27, 28). Protein expression analysis of the copper transporter and cisplatin receptor CTR1 or RNA levels of the multidrug resistance ABC transporters showed no substantial differences in nonexpressing vs. ANGPTL4-overexpressing HNSCC cells (*SI Appendix*, Fig. S1 *A* and *B*) (44, 45). We therefore explored whether ANGPTL4 influenced instead the cisplatin-induced DNA damage response in this cancer. To this end, we ran neutral comet assays to analyze single-cell DNA damage (double-strand breaks) in our HNSCC cell lines cultured in the presence of cisplatin. Interestingly, when we quantified using OpenComet software the tail DNA percentage, tail DNA length, and olive tail moment of CAL27 and CAL27 A4 KO cells treated with the drug, we observed that the DNA damage in CAL27 A4 KO was higher than in the parental CAL27 (Fig. 2 *A*–*D*). Similar results were observed in HN13 A4 KO cells compared to HN13 treated with cisplatin (Fig. 2 *E*–*H*). In agreement with these results, cisplatin-treated CAL27 A4 KO showed increased levels of (Ser^139^) phosphorylated H2AX (γ-H2AX), a molecular marker of DNA damage (DNA double-strand breaks, DSBs), when compared with CAL27 (Fig. 2 *I* and *J*) (46). Conversely, levels of γ-H2AX were lower in HN4 A4 compared to HN4 (Fig. 2 *K* and *L*). These results were corroborated by immunofluorescence in these same cell lines (Fig. 2 *M*–*P* and *SI Appendix*, Fig. S1 *C* and *D*). Extrachromosomal homologous recombination (HR) repair assays in CAL27 A4 KO and HN4 A4 (compared to their respective parental lines) demonstrated that ANGPTL4 promotes repair of DNA DSBs through HR (Fig. 2 *Q* and *R*). Collectively, these findings demonstrate that ANGPTL4 expression in HNSCC promotes DNA damage response and homologous recombination repair in response to cisplatin.

### ANGPTL4 Increases RAD51 Tyr^315^/Tyr^54^ Phosphorylation through NRP1/ABL-1.

We next set out to determine whether ANGPTL4 expression in HNSCC induces cisplatin-initiated HR by regulating the activity of the specific proteins involved in this DDR mechanism. In this regard, RAD51 is a DNA recombinase with critical functions in HR, including the formation of nucleoprotein filaments on single-stranded DNA to catalyze homology search and strand exchange between the ssDNA and a homologous double-stranded DNA (47). RAD51 has been reported to contribute to the reduction of cisplatin-induced DNA damage and its inhibition leads to chemosensitization to the drug (48). We first examined whether RAD51 protein levels were altered upon ANGPTL4 overexpression; however, we observed that RAD51 expression was similar in CAL27 A4 KO and CAL27 in the presence of cisplatin (*SI Appendix*, Fig. S2 *A*–*C*). Similarly, immunofluorescence-based analysis and quantification of RAD51 levels in NOKSI showed no differences in RAD51 expression following treatment with rhANGPTL4, with or without drug exposure (*SI Appendix*, Fig. S2 *D* and *E*).

**Fig. 3.**
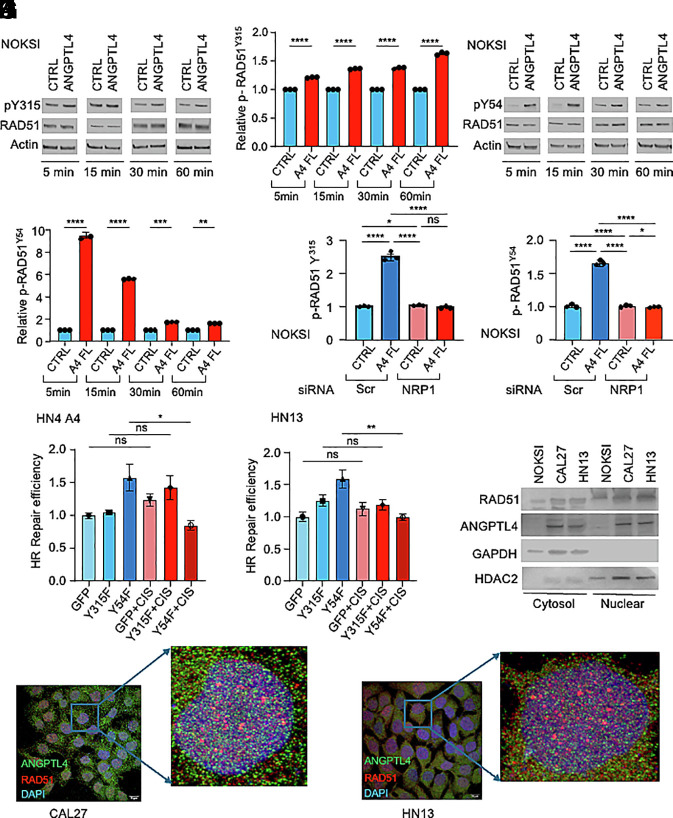
ANGPTL4 increases RAD51 Y54/Y315 phosphorylation. (*A*–*D*) Western blot and densitometric analysis of RAD51 Y315 phosphorylation (*A* and *B*) and RAD51 Y54 phosphorylation (*C* and *D*) upon treatment of NOKSI with rhANGPTL4 for 5, 15, 30, and 60 min. (*E* and *F*) Densitometry of (siRNA-mediated) NRP1 knockdown on RAD51 Y315 phosphorylation (*E*) and RAD51 Y54 phosphorylation (*F*) in NOKSI cells treated with (5 µg/mL) rhANGPTL4. Scr, scrambled siRNA. (*G* and *H*) Relative efficiency of extrachromosomal homologous recombination repair in HN4 A4 (*G*) and HN13 (*H*) upon (6 µg/mL) cisplatin treatment, in cells expressing RAD51 Y315F or RAD51 Y54F mutants. (*I*) Immunoblots of cellular fractionation analysis showing endogenous ANGPTL4 and HR repair protein RAD51 in cytosolic and nuclear fractions of normal NOKSI, CAL27, and HN13. GAPDH (cytosol) and HDAC2 (nuclear) were used as control proteins. (*J* and *K*) Immunofluorescence staining confirming cytoplasmic and nuclear localization of endogenous ANGPTL4 (green) and RAD51 (red) in CAL27 (*J*) or HN13 (*K*). (Scale bar, 15 µm.) Nuclei were counterstained with DAPI (blue). Data are presented as mean ± SEM. ns *P* > 0.05, *P < 0.05, **P < 0.01, ***P < 0.001, and ****P < 0.0001.

Alternatively, RAD51 activity can be regulated by protein phosphorylation. Specifically, sequential phosphorylation of RAD51 on Tyr^315^ and Tyr^54^ by the ABL proto-oncogene 1 nonreceptor tyrosine kinase (ABL1) increases RAD51 strand exchange activity, promoting DDR (49–51). We have previously observed that ANGPTL4 activates ABL1 through a signaling pathway mediated by NRP1 in HNSCC (8). We therefore set out to investigate whether NRP1/ABL1 could be a downstream effector of ANGPTL4-mediated cisplatin chemoresistance, acting on RAD51. Interestingly, we found that treatment of NOKSI with (5 μg/mL) rhANGPTL4 led to mild phosphorylation of RAD51 in Tyr^315^ (Fig. 3 *A* and *B*) and robust phosphorylation in Tyr^54^ (Fig. 3 *C* and *D*). RAD51 Tyr^315^ phosphorylation is important for proper orientation of RAD51 Tyr^54^ into the ABL1 tyrosine kinase domain; Tyr^54^ phosphorylation then enhances RAD51 recombinase activity (49, 51). Interestingly, inhibition of NRP1 expression with a specific NRP1 siRNA led to a reduction in the phosphorylation of these two RAD51 amino acids in NOKSI (Fig. 3 *E* and *F*).

We further investigated the functional relevance of these RAD51 posttranslational modifications by generating RAD51^Y315F^ and RAD51^Y54F^ mutants using site-directed mutagenesis and examining extrachromosomal HR assays upon their expression in our HNSCC cell lines. Expression of RAD51^Y54F^—but not RAD51^Y315F^—was able to inhibit HR in both HN4 A4 and HN13 (Fig. 3 *G* and *H*). Cell lysis and fractionation experiments in NOKSI, CAL27, and HN13 showed that endogenous ANGPTL4 is present in both cytoplasmic and nuclear compartments (Fig. 3*I*). These findings were confirmed using confocal analysis also in CAL27 and HN13 (Fig. 3 *J* and *K*). Collectively, these results suggest that ANGPTL4/NRP1 regulate RAD51 recombinase by increasing its phosphorylation in Tyr^315^/Tyr^54^.

### Inhibition of NRP1 Reverses ANGPTL4-Mediated Increase in DDR and HRR in HNSCC.

**Fig. 4.**
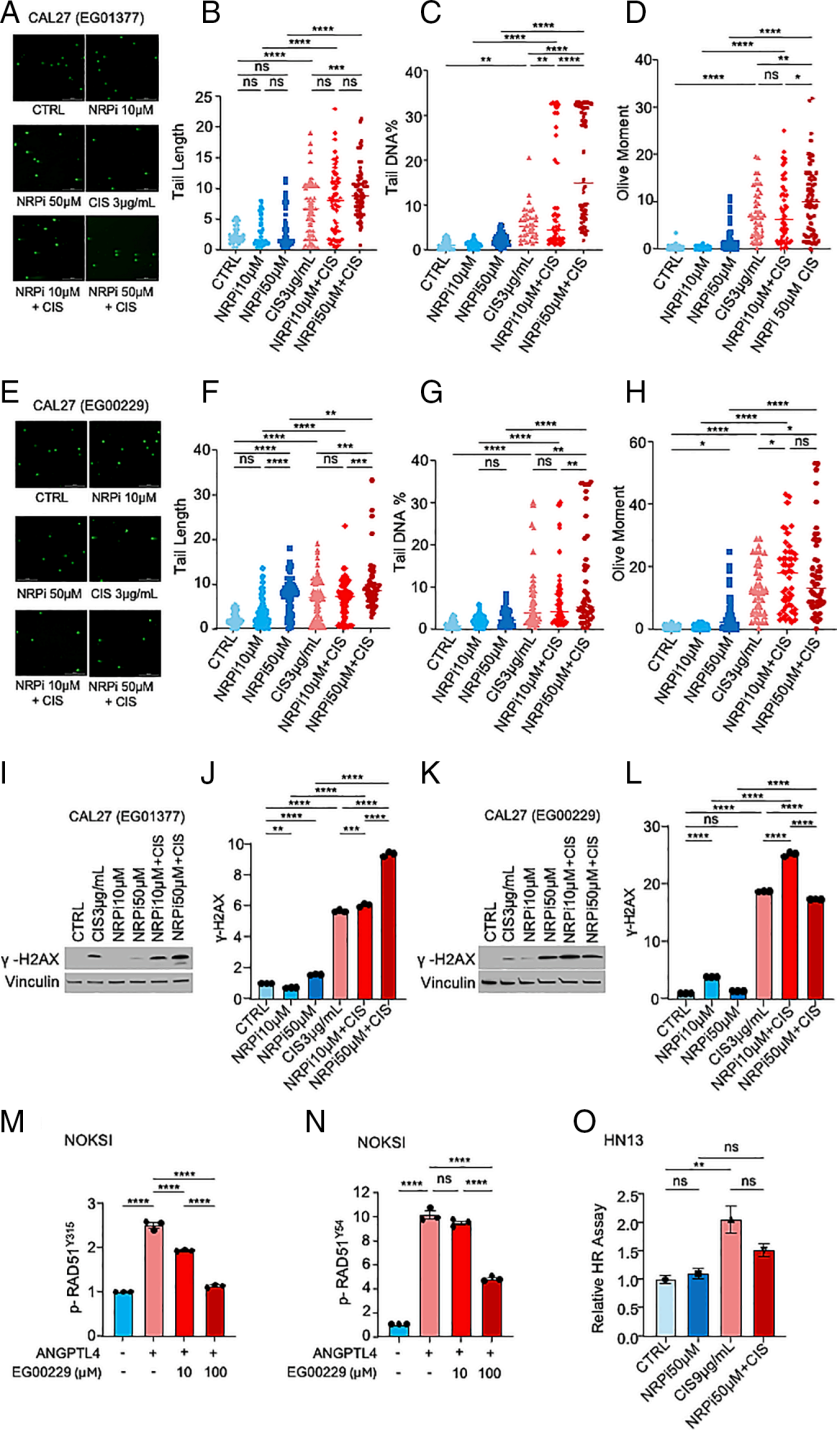
Inhibition of NRP1 reverses ANGPTL4-mediated increase in DNA damage response and homologous recombination repair in HNSCC. (*A*–*D*) Representative pictures of comets (*A*) from neutral comet assay (for DNA DSB) in CAL27 cells treated with (3 µg/mL) cisplatin and NRP1 inhibitor EG01377 (10 µM and 50 µM) for 48 h. Comet parameters: tail length (*B*), tail DNA percentage (*C*), and olive moment (*D*) are provided. (Scale bar, 200 µm.) DNA stained with SYBR green. (*E*–*H*) Representative pictures of comets (*E*) from neutral comet assay (for DNA DSB) in CAL27 cells treated with (3 µg/mL) cisplatin and NRP1 inhibitor EG00229 (10 µM and 50 µM) for 48 h. Comet parameters: tail length (*F*), tail DNA percentage (*G*), and olive moment (*H*) are provided. (Scale bar, 200 µm.) DNA stained with SYBR green. (*I* and *J*) Immunoblot (*I*) and densitometric (*J*) analysis of DNA damage marker γ-H2AX in CAL27 following 24 h treatment with (3 µg/mL) cisplatin and (10 µM and 50 µM) EG01377. (*K* and *L*) Immunoblot (*K*) and densitometric (*L*) analysis of DNA damage marker γ-H2AX in CAL27 cells following 24 h treatment with (3 µg/mL) cisplatin and EG00229 (10 µM and 50 µM). (*M* and *N*) Densitometry of the effect of NRP1 inhibitor EG00229 (10 µM and 100 µM) on RAD51 Y315 phosphorylation (*M*) and RAD51 Y54 phosphorylation (*N*) in NOKSI cells treated with (5 µg/mL) rhANGPTL4 (A4 FL) for 5 min. (*O*) Relative efficiency of extrachromosomal homologous recombination repair in HN13 cells treated with 9 µg/mL cisplatin and EG00229 50 µM for 24 h. Data are presented as mean ± SEM. ns *P* > 0.05, *P < 0.05, **P < 0.01, ***P < 0.001, and ****P < 0.0001.

To further interrogate the mechanism whereby ANGPTL4 promotes DDR and HR, we took advantage of the availability of two NRP1 small molecule inhibitors: EG01377 and EG00229 (52, 53). We treated HNSCC cell lines with EG01377 (Fig. 4 *A*–*D*) or EG00229 (Fig. 4 *E*–*H*) and performed neutral comet and extrachromosomal HR assays in the absence or presence of increasing doses of cisplatin. Interestingly, we found that inhibition of NRP1 with anti-NRP1 drugs led to an increase in the DDR in CAL27 cells (Fig. 4 *A*–*H*). Similar results were found in the other ANGPTL4-overexpressing cell line, HN13 (*SI Appendix*, Fig. S3 *A*–*D*). In agreement with these results, levels of the DDR marker γ-H2AX were increased when NRP1 signaling was blocked in CAL27 or HN13 (Fig. 4 *I*–*L* and *SI Appendix*, Fig. S3 *E* and *F*). Furthermore, pretreatment of NOKSI with EG00229 also blocked ANGPTL4-induced RAD51 phosphorylation in Tyr^315^ and Tyr^54^ (Fig. 4 *M* and *N*). When we examined the effect of pharmacologically targeting NRP1 on HR, we observed that NRP1 inhibition led to a decreased ability of HNSCC cells to repair cisplatin-induced DNA damage through this mechanism (Fig. 4*O*). Collectively, these results suggest that the ANGPTL4-dependent regulation of DDR and HR upon cisplatin treatment in HNSCC is mediated by an autocrine ANGPTL4/NRP1-dependent pathway.

### Inhibition of ABL1 Reverses ANGPTL4-Mediated Increase in DDR and HRR in HNSCC.

**Fig. 5.**
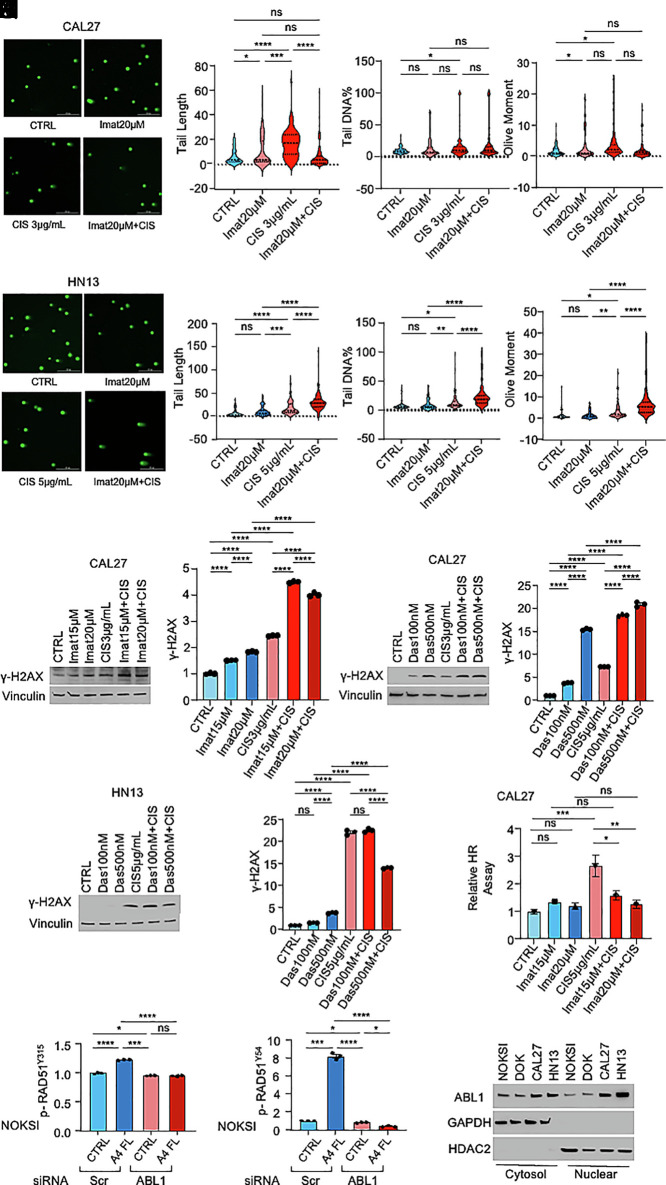
Inhibition of ABL1 reverses ANGPTL-4-mediated increase in DNA damage response and homologous recombination repair in HNSCC. (*A*–*D*) Representative pictures of comets (*A*) from neutral comet assay (for DNA DSB) in CAL27 cells treated with (3 µg/mL) cisplatin and ABL1 inhibitor (20 µM) imatinib for 48 h. Comet parameters: tail length (*B*), tail DNA percentage (*C*), and olive moment (*D*) are provided. (Scale bar, 200 µm.) DNA stained with SYBR green. (*E*–*H*) Representative pictures of comets (*E*) from neutral comet assay (for DNA DSB) in HN13 cells treated with (5 µg/mL) cisplatin and ABL1 inhibitor (20 µM) imatinib for 48 h. Comet parameters: tail length (*F*), tail DNA percentage (*G*), and olive moment (*H*) are provided. (Scale bar, 200 µm.) DNA stained with SYBR green. (*I* and *J*) Immunoblot (*I*) and densitometric (*J*) analysis of DNA damage marker γ-H2AX levels in CAL27 cells following 24 h treatment with (3 µg/mL) cisplatin and ABL1 inhibitor dasatinib (100 nM and 500 nM). (*K* and *L*) Immunoblot (*K*) and densitometric (*L*) analysis of DNA damage marker γ-H2AX levels in CAL27 cells following 24 h treatment with (3 µg/mL) cisplatin and imatinib (15 µM and 20 µM). (*M* and *N*) Immunoblot (*M*) and densitometric (*N*) analysis of DNA damage marker γ-H2AX levels in HN13 cells treated with (5 µg/mL) cisplatin and dasatinib (100 nM and 500 nM) for 24 h. (*O* and *P*) Densitometry of (siRNA-mediated) ABL1 knockdown on RAD51 Y315 phosphorylation (*O*) and RAD51 Y54 phosphorylation (*P*) in NOKSI cells treated with (5 µg/mL) rhANGPTL4 for 5 min. Scr, scrambled siRNA. (*Q*) Relative efficiency of extrachromosomal homologous recombination repair in CAL27 cells treated with (5 µg/mL) cisplatin and imatinib (15 µM and 20 µM) for 24 h. (*R*) Immunoblots of cellular fractionation analysis showing endogenous ABL1 in cytosolic and nuclear fractions of NOKSI, dysplastic oral keratinocytes (DOKSI), CAL27, and HN13 cells. Cytosolic (GAPDH) and nuclear (HDAC2) proteins were used as controls. Data are presented as mean ± SEM. ns *P* > 0.05, *P < 0.05, **P < 0.01, ***P < 0.001, and ****P < 0.0001.

We next investigated whether pharmacological inhibition of ABL1 could prevent ANGPTL4-induced decreased sensitivity to cisplatin in HNSCC. To this end, we used two FDA-approved ABL1 inhibitors, Imatinib and Dasatinib (54–56). Interestingly, we observed an inhibition of DDR (analyzed by neutral comet assay) with treatment of CAL27 or HN13 with (20 μM) Imatinib (Fig. 5 *A*–*H*). Pharmacological inhibition of ABL1 with Imatinib similarly led to an increase in γ-H2AX in CAL27 (Fig. 5 *I* and *J*). Similar results were observed in CAL27 and HN13 treated with (100 nM or 500 nM) Dasatinib (Fig. 5 *K*–*N*). Expression of a specific ABL1 siRNA in NOKSI was able to block RAD51 phosphorylation on Tyr^315^ and Tyr^54^ (Fig. 5 *O* and *P*). Accordingly, the pharmacological inhibition of ABL1 with (20 μM) Imatinib was able to block the ability of HNSCC cells to undergo HR following treatment with cisplatin (Fig. 5*Q*). Cellular fractionation studies confirmed ABL1 is present in the cytosolic and nuclear fractions of our HNSCC cell lines, similar to ANGPTL4 and RAD51 (Fig. 5*R*). Collectively, these results suggest that the ANGPTL4-dependent regulation of DDR and HR in response to cisplatin in HNSCC is dependent on ABL1.

### Inhibition of NRP1 or ABL1 Markedly Increases Chemosensitization to Cisplatin in HNSCC In Vitro and In Vivo.

**Fig. 6.**
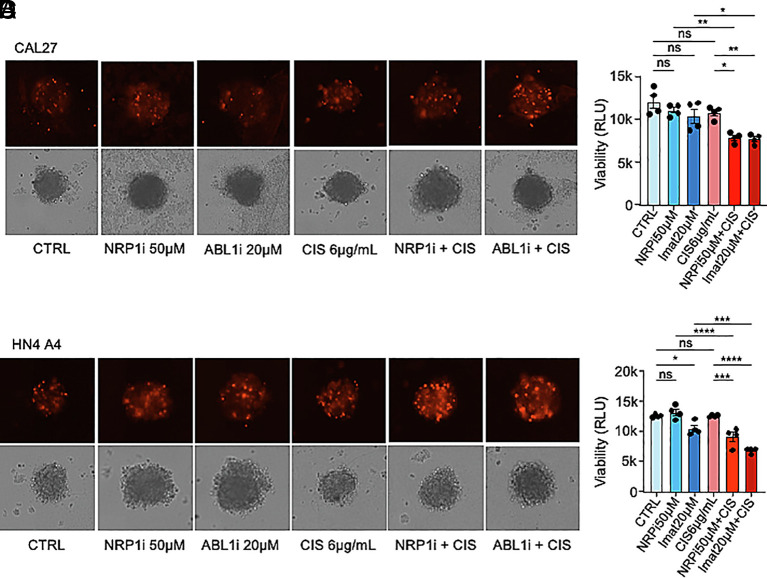
Inhibition of NRP1 and ABL1 sensitizes HNSCC cell spheroids to cisplatin. (*A* and *B*) Brightfield and fluorescent micrographs (PI staining, red) (*A*) and viability determined with Cell Titer Glo assay (*B*) of CAL27 spheroids treated with EG00229 (NRP1i, 50 µM), imatinib (ABL1i, 20 µM), cisplatin (6 µg/mL), and combinations. (*C* and *D*) Brightfield and fluorescent micrographs (PI staining, red) (*C*) and viability determined with Cell Titer Glo assay (*D*) of HN4 A4 spheroids treated with EG00229 (NRP1i, 50 µM), imatinib (ABL1i, 20 µM), cisplatin (6 µg/mL), and combinations. Data are presented as mean ± SEM. ns *P* > 0.05, *P < 0.05, **P < 0.01, ***P < 0.001, and ****P < 0.0001.

Our results suggest that ANGPTL4/NRP1/ABL1/RAD51 may be an effective target for chemosensitization to cisplatin in HNSCC and that therapies targeting this pathway could have an impact in the treatment of this aggressive cancer. We therefore examined the effects of combinatorial treatments using cisplatin along with inhibition of NRP1 or ABL1 in our HNSCC cell lines. Interestingly, when we treated CAL27 or HN13 with sub-IC_50_ (3 µg/mL) cisplatin and (10 µM or 50 µM) EG01377 or (10 µM or 50 µM) EG00229 and quantified tumor cell apoptosis, we found that the combination of drugs was more effective at inducing cell death than either treatment alone (*SI Appendix*, Fig. S4 *A*–*D*). We also quantified the levels of apoptosis upon treatment of CAL27 cells with cisplatin (3 µg/mL) along with siRNA-mediated knockdown of ABL1 (*SI Appendix*, Fig. S4*E*) or (100 nM or 500 nM) Dasatinib (*SI Appendix*, Fig. S4*F*) and observed an increase in the percentage of apoptotic cells. Similar results were observed with HN13 cells (*SI Appendix*, Fig. S4 *G* and *H*). We next determined the cell viability of CAL27 or HN4 A4 3D tumor spheroids exposed to sub-IC_50_ concentrations of cisplatin along with either EG00229 or Imatinib, using Cell Titer Glo analysis. In agreement with the previous results, we found a decrease in cell viability when the two drugs were used in combination compared to either drug alone (Fig. 6 *A*–*D*).

**Fig. 7.**
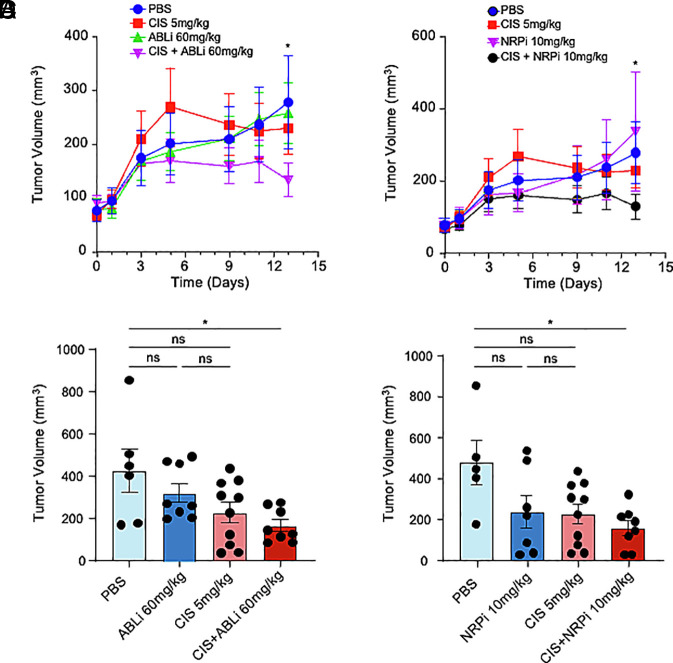
Inhibition of NRP1 and ABL1 sensitizes HNSCC to cisplatin in vivo. (*A* and *B*) In vivo response to cisplatin, imatinib (ABLi) or combination of both (*A*) or cisplatin, EG00229 (NRPi) or combination of both (*B*) in CAL27 xenografts generated upon subcutaneous injection of 2 × 10^6^ CAL27 in both flanks of (nu/nu) mice (n = 5). Drugs were administered intraperitoneally. Tumor burden of resulting xenografts (n = 8/10) is shown. (*C* and *D*) Tumor volume of CAL27 xenografts treated with cisplatin, imatinib (ABLi) or combination of both (*C*) or cisplatin, EG00229 (NRPi) or combination of both (*D*) at the end of treatment. Data are presented as mean ± SEM. ns *P* > 0.05, *P < 0.05, **P < 0.01, ***P < 0.001, and ****P < 0.0001.

To assess these effects in vivo, we generated HNSCC xenografts by injecting CAL27 subcutaneously in the flanks of immunocompromised mice. Treatment of generated CAL27 xenografts with imatinib (60 mg/kg; Fig. 7*A*) or EG00229 (10 mg/kg; Fig. 7*B*), at doses which did not influence tumor growth alone, sensitized CAL27 to a subtherapeutic dose of cisplatin (5 mg/kg) markedly reducing tumor volume respectively (Fig. 7 *C* and *D*). Tumor microvessel density of the combinatorial treatments was modestly reduced but did not reach statistical significance for EG00229 (*SI Appendix*, Fig. S5 *A* and *B*). This suggests that the previously reported antiangiogenic properties of therapies targeting ANGPTL4 (21) are not sufficient to explain the additive effects of combining treatments targeting ANGPTL4 signaling with cisplatin. Collectively, these findings support a role for the ANGPTL4/NRP1/ABL1 axis in facilitating cisplatin chemosensitization in HNSCC.

## Discussion

ANGPTL4 and other members of the ANGPTL family are multifunctional proteins involved in biological processes more disparate than the ones of their structurally related homologs, ANGPTs (57, 58). In addition to ANGPTL4’s critical roles regulating lipid and carbohydrate metabolism, energy fuel use and storage, and dysregulated neovascularization and hyperpermeability, compelling evidence suggests that this adipokine is an important tumor biomarker with autocrine protumorigenic functions in cancer cells. ANGPTL4 overexpression is associated with poor clinical outcomes in tumors with adverse clinicopathological features including breast cancer, ovarian cancer, uveal melanoma, colon cancer, gastric cancer, and HNSCC (7–9, 59–64). New discoveries into the specific signaling networks regulated by ANGPTL4 in cancer may bring fundamental insight into the elucidation of putative therapeutic avenues targeting this factor and its signaling partners for patients with this disease.

Here, we provide evidence of the role of ANGPTL4 in platinum-based chemoresistance through the promotion of DDR and HR in HNSCC tumor cells. Correlation of ANGPTL4 expression and decreased cisplatin chemosensitivity was found in a set of HNSCC PTDOs, HNSCC xenografts, as well as in tumor spheroids and cell cultures of established HNSCC cell lines. Experiments revealed that ANGPTL4-dependent loss of sensitivity to the drug and decreased tumor cell apoptosis was caused by an increase in RAD51 Tyr^315/54^ phosphorylation and enhanced HR and DDR, through an NRP1/ABL1-dependent pathway. RAD51 recombinase is a crucial HR protein that facilitates the homology search and strand exchange to repair DSBs. It polymerizes onto the single-stranded DNA to form a nucleoprotein filament, with the aid of the single-stranded DNA–binding protein, replication protein A (RPA), and RAD52 (65). The recombinase activity of RAD51 and its effects in DDR are regulated by its association with proteins including BRCA1, BRCA2, and several RAD51 paralogues and by protein phosphorylation. In this regard, compelling data have shown that ABL1-dependent phosphorylation of RAD51 in Tyr^315^ and Tyr^54^ occurs sequentially and that Tyr^315^ phosphorylation derives in a RAD51 conformational change that facilitates its phosphorylation in Tyr^54^. Interestingly, we found a moderate ANGPTL4-mediated phosphorylation of RAD51 Tyr^315^ and a profound effect in the phosphorylation of Tyr^54^, a pivotal amino acid for RAD51 function (66). Tyr^54^ phosphorylation enhances the RAD51 recombinase activity by at least two different mechanisms: modifying the RAD51 nucleoprotein filament itself and allowing RAD51 to compete efficiently with ssDNA binding protein RPA (49, 51). Ultimately, enhanced RAD51 response leads to increased HR and cell survival in drug-treated cells.

Some studies had indicated the role of ANGPTL4 in cancer platinum-based chemoresistance; however, the mechanism and translational significance of this effect remained poorly understood (67, 68). In our studies on ANGPTL4-dependent HR, we did not observe any changes in RAD51 expression, formation of RAD51 nucleofilament foci, or extensive changes in Tyr^315^ (also thought to be involved in RAD51 polymerization and foci formation). Our imaging and cell fractionation/immunoblot analysis showed that ANGPTL4, acting on HNSCC cells in an autocrine manner, is present both in the cytoplasmic and the nuclear compartments in HNSCC cells, which is consistent with previous findings on ANGPTL4 localization in triple-negative breast cancer (TNBC) cell lines (69). Previous evidence suggests that RAD51 phosphorylation affects the nuclear translocation of RAD51 within the cell in response to DNA damage (70). Whether this occurs upon ANGPTL4/NRP1/ABL1 activation is still unknown. Additionally, we did not find any changes in the expression of the cisplatin receptor CTR1 or the multidrug resistance ABC transporters suggesting no changes in cisplatin uptake or export. These results agree with others previously reported in the literature. Indeed, bioinformatic analysis of 565 patient data from the Cancer Genome Atlas (TCGA) revealed that cisplatin uptake proteins CTR1 and OCT1 were not significantly altered in patients who had no residual HNSCC tumors vs. patients who had HNSCC residual tumors (71).

Our studies are relevant for tumors that show upregulated expression of ANGPTL4, many of which face the burden of platinum-based chemoresistance. As for HNSCC, previous studies from our laboratory have revealed that ANGPTL4 and its signaling effectors are key biomarkers in HNSCC progression and dissemination and may be putative therapeutic targets for HNSCC management (8, 9). Although the clinical outcomes of HNSCC patients with early-stage disease (stage I or stage II) are satisfactory (5-y overall survival of 70 to 90%), more than 60% of HNSCC patients are diagnosed at advanced stages of the disease (stage III or stage IV) and have high probability of recurrence and metastatic disease. These patients require combined chemotherapy and chemoradiotherapy protocols and cisplatin, 5-fluorouracil (5FU), paclitaxel (PTX), or docetaxel (DTX) as the most used chemotherapeutic agents (37). Unfortunately, treatment failure and/or chemotherapy resistance often arise. Approved immunotherapy against EGFR has shown modest advantage in HNSCC (39). PD-1/PD-L1 inhibitors such nivolumab, pembrolizumab, and dostarlimab appear to increase overall survival (OS) and progression-free survival (PFS) and are used clinically, particularly for patients with poor prognoses and refractory to treatment. Nevertheless, patient median survival time remains within a range of 1 to 2 y for advanced HNSCC (72). Here, we show data that support another important role of ANGPTL4 as an HNSCC molecular and therapeutic target. Besides ANGPTL4, NRP1 is also a molecular marker associated with HNSCC poor prognosis (8, 73–75). Inhibiting ANGPTL4 signaling routes may be advantageous for combinatorial treatments for HNSCC patients and other cancer showing upregulation of these proteins.

Furthermore, these findings support the recent appreciation of angiogenic factors in roles beyond their vasoactive functions (76). The HIF effector, VEGF, has been shown to induce YAP/TAZ-mediated RAD51 transcription in TNBC, independently of its role in angiogenesis (77). ANGPTL8 was recently reported to localize to the nucleus and regulate DNA damage response and apoptosis in hepatic cancer cell lines HepG2 and Hep3B (78). NRP1 knockdown in a VEGFR2-inhibited background also upregulated the expression of RAD51 protein in non–small cell lung cancer cells to favor radioresistance (79). All this supports a more complex involvement of angiogenic proteins in many distinct aspects of cancer development.

Both VEGF and ANGPTL4 share the capacity of signaling through NRP1 and NRP2. NRP inhibitors are under study for the management for different disparate diseases (80, 81). Our data suggest they could be repurposed to counteract resistance to cisplatin in HNSCC and other cancers with elevated ANGPTL4 and/or VEGF expression. Whether a dual NRP1/NRP2 inhibitor might be more effective for this purpose to combat HNSCC is under investigation. Furthermore, ANGPTL4-phosphorylation of RAD51 shows to be dependent on ABL1 and that is also valuable as several inhibitors of this kinase are already FDA-approved and used in cancer chemotherapy protocols. Much debate is happening regarding the advantages of second and third generation of ABL1 inhibitors with respect to imatinib as the new drugs appear to be more toxic and do not have clear survival benefits, at least for chronic myeloid leukemia (CML) (82, 83). The effects of ABL1 inhibitors for inhibition of RAD51 and HR surely warrants further investigation.

## Methods

### Cell Culture and Reagents.

Normal oral keratinocytes (NOKSI) were grown in keratinocyte serum-free media supplemented with growth factors (Gibco) and 1% penicillin-streptomycin. Dysplastic oral keratinocytes (DOK) grew in DMEM with 10% FBS, 0.05% hydrocortisone, and 1% penicillin-streptomycin. HNSCC cell lines HN13, HN4, and CAL27 were cultured in DMEM with 10% FBS and 1% penicillin-streptomycin. Human full-length ANGPTL4 protein was obtained from R&D Systems. Cisplatin and Imatinib were obtained from Millipore-Sigma; EG00229 trifluoroacetate, EG01377 dihydrochloride from MedChemExpress, and Dasatinib from Tocris Bio-Techne.

### CRISPR-Mediated Knockout Gene, siRNA, and cDNA Expression.

CAL27 A4 KO and HN13 A4 KO were generated using CRISPR Cas9 by the UMB Translational Laboratory Shared Service. (Qiagen) siRNA or ectopic ANGPTL4 (pcDNA3.1-ANGPTL4-mycHis) expression was achieved using the Nucleofector™ kit (Amaxa Biosystems).

### Western Blot Analysis and Immunofluorescence.

Western blot analysis was performed as in ref. 8, and immunofluorescence was performed as in ref. 84.

### Cell Viability Assays: Crystal Violet, MTT, and Annexin V/PI Assay.

The efficacy of cisplatin in cells was determined using the crystal violet assay kit (Abcam), 3-[4,5-dimethylthiazole-2-yl]-2,5-diphenyltetrazolium bromide (MTT) assay kit (Abcam), and Dead Cell Apoptosis kit with Annexin V FITC and Propidium iodide kit for flow cytometry (Invitrogen).

### Neutral Single-Cell Gel Electrophoresis or Comet Assay.

For the detection of double-strand break DNA damage, the neutral version of the comet assay was performed according to R&D Systems specifications (4250-050-K).

### In Vitro Extrachromosomal Homologous Recombination Repair Efficiency Assay.

Extrachromosomal HR assay was performed according to ref. 2.

### HNSCC Tumor-Derived Organoid Generation and Characterization.

HNSCC tumor-derived organoid generation, culture, and genomic characterization were already described in ref. 41.

### Tumor Spheroid Culture, Imaging, and Viability Assay.

For qualitative studies, (3D) tumor spheroids were created using low-attachment culture plates for 96 h and imaged with Celigo Imaging Cytometer. For quantitative studies, the RASTRUM Platform was used according to ref. 85.

### ABC Transporters Assay, RNA Extraction, and cDNA Synthesis.

Gene expression of human ABC transporters was profiled using the Human ABC Transporters TaqMan™ Array Fast 96-well (Thermo Fisher Scientific Cat# 4418811). Raw Cq-values obtained were used to calculate the fold change (2 ^−ΔΔC^_t_).

### Subcellular Protein Fractionation.

Subcellular protein fractionation was performed using a kit from ThermoFisher Scientific (#78840).

### Site-Directed Mutagenesis.

Site-directed mutagenesis (Tyr 54 and Tyr 315) was performed using the site-directed Quick Change II XL Site-Directed Mutagenesis Kit from Agilent Technologies (#200521). CMV-hRAD51 plasmid containing RAD51 insert (125570) was used as the template.

### Mouse Xenografts Assays.

Athymic (nu/nu) 8-wk-old female nude mice were supplied by Jackson (Jax®) Laboratories. All animal experiments were carried out after review and approval by the Johns Hopkins Institutional Animal Care and Use Committee. Experiments were performed as in ref. 8. The treatments were administered intraperitoneally at the following doses: 1) 5 mg/kg cisplatin, 2) 2 mg/kg EG00229, 3) 10 mg/kg EG00229, 4) 60 mg/kg Imatinib, 5) 5 mg/kg cisplatin and 2 mg/kg EG00229, 6) 5 mg/kg cisplatin and 10 mg/kg EG00229, 7) 5 mg/kg cisplatin and 60 mg/kg Imatinib, and 8) PBS or control. Tumor volume was reported as $\mathrm{Volume}=\frac{\mathrm{Length}*{\text{Width}}^{2}}{2}$. Microvessel density was determined as in ref. 21.

### Statistics.

Statistical analyses were conducted using the GraphPad Prism 10.0 statistical software. Data were expressed as mean ± SEM from at least 3 independent experiments. To determine statistically significant differences between test groups, unpaired Student’s *t* test, one-way and two-way ANOVA with follow-up Bonferroni post hoc test. The *t* test was used for analyzing differences between two groups and one- and two-way ANOVA for more than two groups followed by post hoc analysis.

## Supplementary Material

Appendix 01 (PDF)

Dataset S01 (XLSX)

Dataset S02 (PDF)

## Data Availability

Previously published data were used for this work (41). All other data are included in the manuscript and/or supporting information.
