# Supplementary material for: Targeting the ANGPTL4/NRP1/ABL1/RAD51 axis reverses cisplatin resistance by impairing DNA damage repair in head and neck cancer
Source: Proc Natl Acad Sci U S A. 2026 Mar 26;123(13):e2510265123. doi: 10.1073/pnas.2510265123 (PMC13038062; doi:10.1073/pnas.2510265123)
Supplement: Supplementary file 1 — Appendix 01 (PDF) [file pnas.2510265123.sapp.pdf]

Supporting information for

Targeting the ANGPTL4/NRP1/ABL1/RAD51 axis reverses cisplatin resistance by impairing DNA damage repair in head and neck cancer

Emmanuel B. Asiedu, Ajay Kumar, Alexander Choi, Derek Osorio Luciano, Kevin Lo, Deepti Sharma, Tao Ma, Feyruz Rassool, Akrit Sodhi, and Silvia Montaner*

*Correspondence:
Silvia Montaner, Ph.D., M.P.H.
Department of Oncology and Diagnostic Sciences, School of Dentistry
Greenebaum Comprehensive Cancer Center
650 W Baltimore St., Room 7263
Baltimore, MD 21201, United States
FAX: 410-706-6115
Email: smontaner@umaryland.edu

This PDF file includes:

Supplemental Figures S1 to S5 with Figures Legends
Supplemental Methods
List of Antibodies
Uncut Gels for Main and Supplemental Figures

SUPPLEMENTAL FIGURES

Supplemental Figure 1

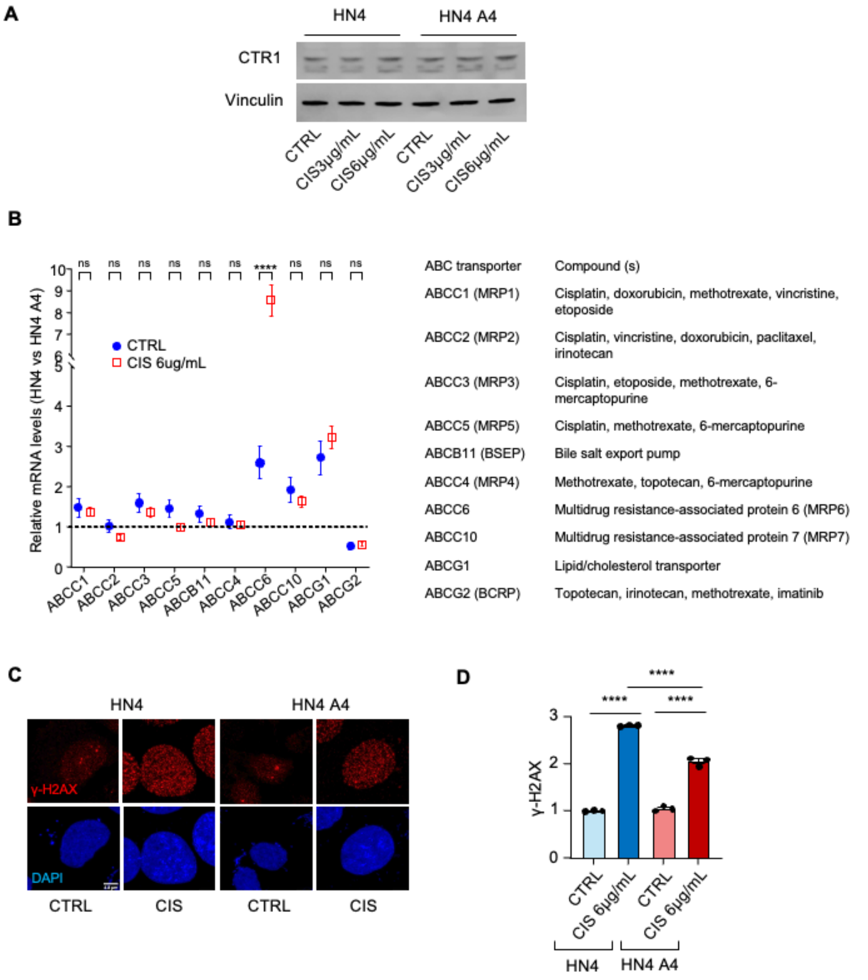

Figure S1: ANGPTL4 does not affect levels of CTR1 or multidrug resistance (MDR) proteins/ABC transporters in HNSCC.

A. Immunoblot analysis reveals no change in cisplatin and copper transporter CTR1 levels in HN4 and HN4 A4 cells following 24hr treatment with (3µg/ml and 6µg/ml) cisplatin. **B.** Fold change in mRNA expression of relevant multidrug resistance (MDR) proteins/ABC transporter proteins in HN4 and HN4 A4 cells with and without cisplatin treatment. Data is presented as expression in HN4 vs expression in HN4 A4 determined by TaqMan^{TM} array (B). **C.** Immunofluorescence staining for γ-H2AX (red) in HN4 and HN4 A4 following 24hr treatment with (6µg/ml) cisplatin. Scale bar, 4.8µm. Nuclei are stained with DAPI. **D.** Quantification of immunofluorescence intensity in (C). Data are presented as mean±SEM. ns $p>0.05$, *$p<0.05$, **$p<0.01$, ***$p<0.001$, and ****$p<0.0001$.

Supplemental Figure 2

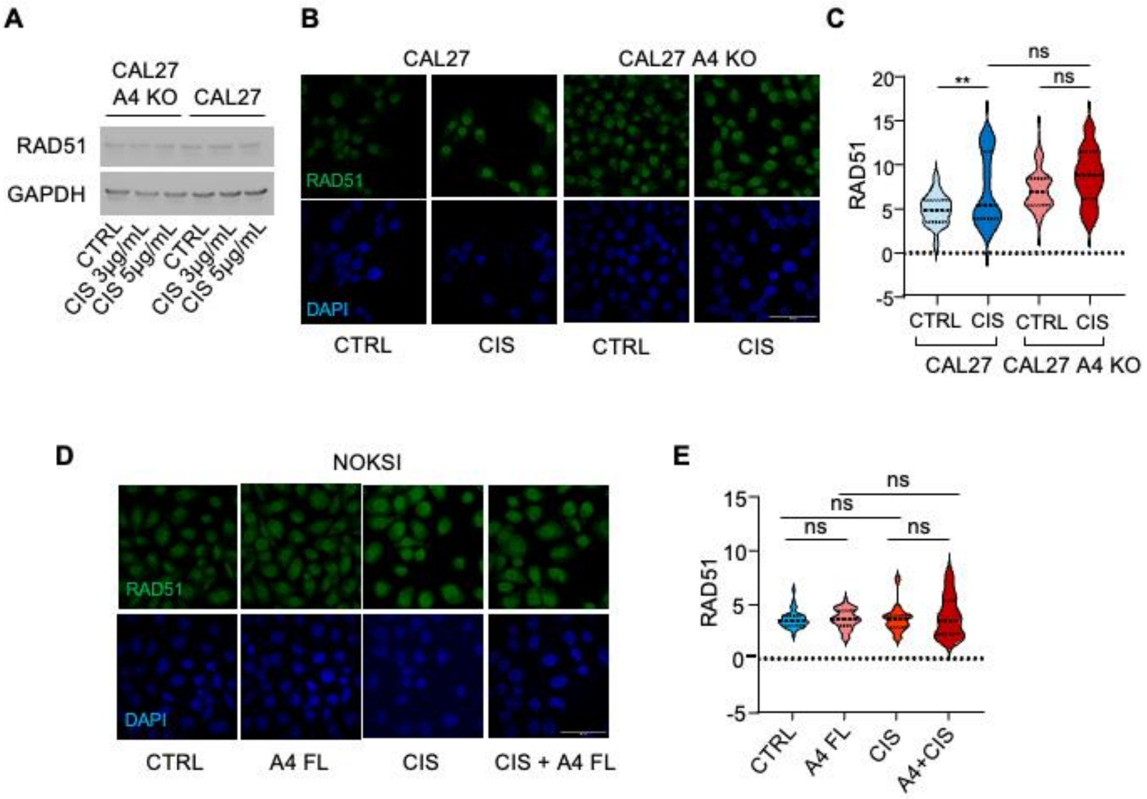

Figure S2: ANGPTL4 upregulation does not affect RAD51 expression levels.
A. Western blot analysis of RAD51 in CAL27 and CAL27 A4 KO cells following 24hr treatment with (3µg/ml and 5µg/ml) cisplatin. **B.** Immunofluorescence staining for RAD51 (green) in CAL27 and CAL27 A4 KO cells treated with 5µg/ml cisplatin for 24hr. Scale bar, 50µm. Nuclei were stained with DAPI. **C.** Quantification of RAD51 immunofluorescence intensity from at least 30 cells in panel B. **D.** Immunofluorescence staining for RAD51 (green) in NOKSI cells treated with (5µg/ml) rhANGPTL4 full length (A4 FL) and (5µg/ml) cisplatin for 24hr. Scale bar, 50µm. Nuclei were stained with DAPI. **E.** Quantification of RAD51 immunofluorescence intensity from at least 40 cells in panel D. Data are presented as mean±SEM. ns $p>0.05$ and $^{**}p<0.01$.

Supplemental Figure 3

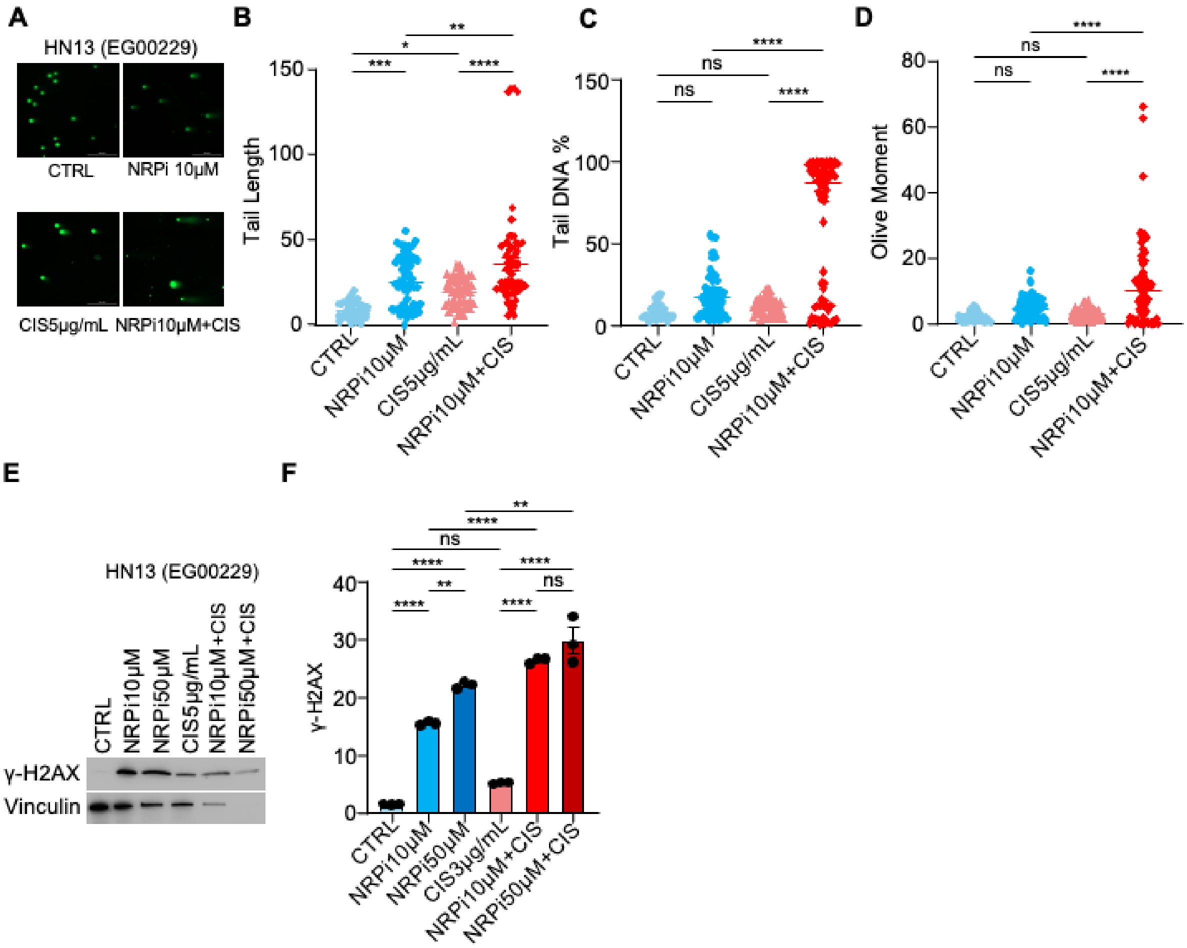

Figure S3: Inhibition of NRP1 reverses ANGPTL4-mediated increase in DNA damage response and homologous recombination repair in HNSCC.

A-D. Representative pictures of comets (A) from neutral comet assay (for DNA DSB) in HN13 cells treated with (5µg/ml) cisplatin and NRP1 inhibitor (10µM) EG00229 for 48hrs. Comet parameters: tail length (B), tail DNA percentage (C), and olive moment (D) are provided. Scale bar, 200µm. DNA stained with SYBR green. **E-F.** Western Blot (E) and densitometric (F) analysis of DNA damage marker γ-H2AX levels in HN13 cells following 24hr treatment with (5µg/ml) cisplatin and EG00229 (10µM and 50µM). Data are represented as mean ± SEM. ns $p>0.05$, *$p<0.05$, **$p<0.01$, ***$p<0.001$, and ****$p<0.0001$.

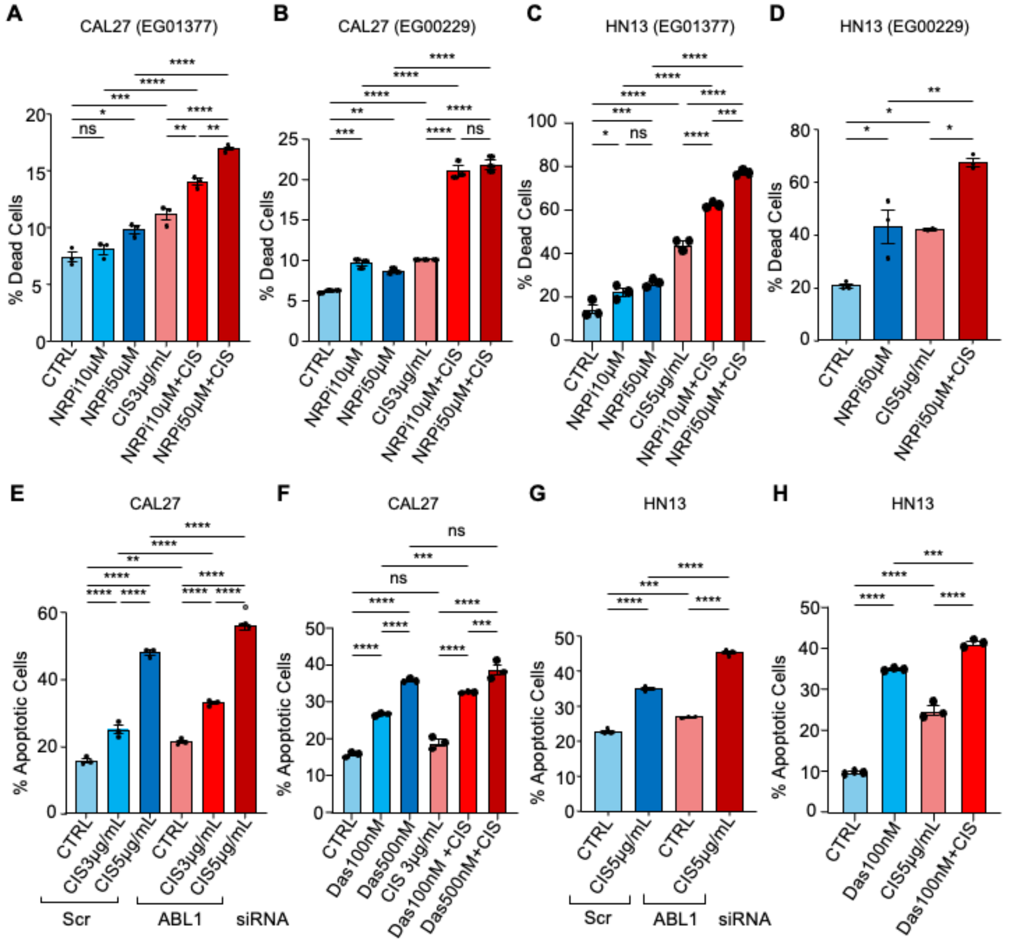

Figure S4: Inhibition of NRP1 and ABL1 sensitizes HNSCC cells to cisplatin

A-B. Percentage of dead cells in CAL27 cultures treated with (3μg/ml) cisplatin and NRP1 inhibitor EG01377 (10μM and 50μM) (A) and EG00229 (10μM and 50μM) (B) for 24hrs determined by annexin V/PI assay and flow cytometry. **C-D.** Percentage of dead cells in HN13 cells treated with (5μg/ml) cisplatin and NRP1 inhibitor EG01377 (10μM and 50μM) (C) and (50μM) EG00229 (D) for 24hrs determined by annexin V/PI assay and flow cytometry. **E.** Percentage of dead cells in CAL27 cells following (siRNA-mediated) knockdown of ABL1 and treated with cisplatin (3μg/ml and 5μg/ml). Scr, scrambled siRNA. **F.** Percentage of dead cells in CAL27 cells treated with (3μg/ml) cisplatin and dasatinib (100nM and 500nM) for 24hrs determined by annexin V/PI assay followed by flow cytometry. **G.** Percentage of dead cells in HN13 cells following siRNA-mediated ABL1 knockdown and treated with (5μg/ml) cisplatin. Scr, scrambled siRNA. **H.** Percentage of dead cells in HN13 cells treated with cisplatin (5μg/ml) and dasatinib (100nM) for 24hrs determined by annexin V/PI assay followed by flow cytometry. Data are presented as mean±SEM. ns $p>0.05$, *$p<0.05$, **$p<0.01$, ***$p<0.001$, and ****$p<0.0001$.

Supplemental Figure 5

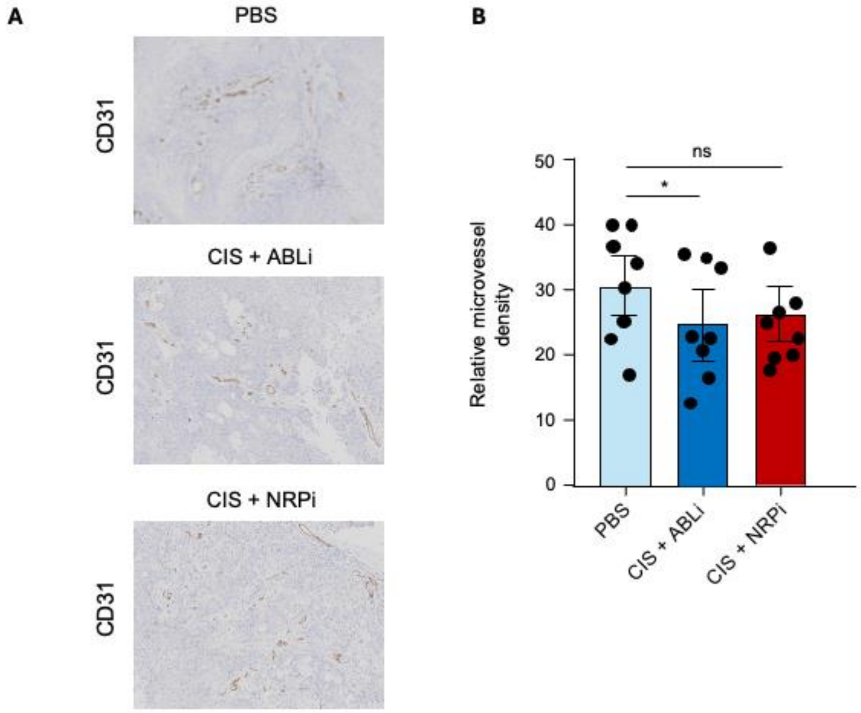

Figure S5: Effect of the dual cisplatin/NRP1 inhibitor or cisplatin/ABL1 inhibitor treatment on HNSCC xenograft microvessel density.

A-B. Representative images (40x) of the CD31 immunohistochemical staining of the CAL27 xenografts treated with PBS, (5 mg/kg) cisplatin and (10 mg/kg) EG00229 (NRPi) or (5 mg/kg) cisplatin and (60 mg/kg) imatinib (ABLi) shown in Figure 7 (A), and determination of their relative microvessel density (B). Data are represented as mean ± SEM. ns $p>0.05$, $^{*}p<0.05$.

SUPPLEMENTAL METHODS

Cell culture and reagents

Human-derived spontaneously immortalized normal oral keratinocytes (NOKSI) were grown in keratinocyte serum free media supplemented with growth factors (Gibco) and 1% penicillin-streptomycin. Dysplastic oral keratinocytes (DOK) from human dorsal tongue grew in DMEM with 10% FBS, 0.05% hydrocortisone, and 1% penicillin-streptomycin. HNSCC cell lines HN13 (tongue – T2N2M0), HN4 (base of tongue – T4N1M0), and CAL27 (tongue) were cultured in DMEM with 10% FBS and 1% penicillin-streptomycin. NOKSI, DOK, HN13 and HN4 were a kind gift from Dr. Abraham Schneider (University of Maryland, Baltimore). CAL27 were obtained from the ATCC. UMB Translational Laboratory Shared Service tested and authenticated all cell lines. Recombinant human full-length ANGPTL4 (A4-FL) protein was obtained from R&D systems. Cisplatin and Imatinib mesylate were obtained from Millipore-Sigma; EG00229 trifluoroacetate, EG01377 dihydrochloride from MedChemExpress, and Dasatinib from Tocris Bio-Techne.

Cytotoxic drug preparations

Cisplatin was dissolved in PBS supplemented with 140mM NaCl. EG00229 and EG01377 were prepared according to manufacturer's instructions, dissolved in DMSO. Imatinib mesylate was dissolved in water. Dasatinib was dissolved in DMSO.

CRISPR-mediated knockout gene, siRNA, and cDNA expression

CAL27 and HN13 ANGPTL4 KO cells were generated using CRISPR Cas9 mechanism with two synthetic single-guide RNAs (sgRNA1 and sgRNA2) by the UMB Translational Laboratory Shared Service (CRISPR core). siRNAs were obtained from Qiagen and expressed in cells using Nucleofector™ kit (Amaxa Biosystems). Stable ectopic expression of ANGPTL4 in HN4 was achieved by nucleofection of pcDNA3.1-ANGPTL4-mycHis with the Lonza Amaxa™ 4D-Nucleofector platform followed by antibiotic selection to generate the HN4 A4 line.

Western blot analysis

Western blot analysis was performed as in (8). Briefly, cells are washed with ice-cold PBS and incubated for 30 minutes in M-PER mammalian protein extraction buffer supplemented with proteinase and phosphatase inhibitor cocktail and ethylene diamine tetraacetic acid (EDTA) (ThermoScientific) at 4ºC. Supernatant containing proteins were retrieved after centrifugation and protein quantification performed using the Bradford assay. Equal amounts of proteins (typically 20µg) were loaded on polyacrylamide gels and separated by sodium dodecyl sulfate-polyacrylamide gel electrophoresis (SDS-PAGE) on the basis on molecular weight or size. For transfer of proteins to PVDF membrane, the Bio-Rad Trans-Blot Turbo transfer system and protocol was used. Membranes with proteins were blocked with 5% milk or bovine serum albumin (BSA) in Tris-buffered saline with Tween 20 and incubated with primary antibodies (typically at 1:1000 dilution) overnight. Blots were then incubated in secondary antibody (typically at 1:5000) and developed with KwikQuant Imager.

Immunofluorescence

Immunofluorescence was performed as in (85). Briefly, HNSCC cells were plated onto sterile coverslips and treated with cisplatin for 24 hours. Cells were then exposed to 4% paraformaldehyde for 10 min to fix, washed 3 times in Dulbecco's phosphate-buffered saline (DPBS), and permeabilized for 10 min in permeabilization buffer. Coverslips were washed 3 times in DPBS + 1% bovine serum albumin + 0.1% Triton X-100, then blocked for 1h RT, washed 3 times, and incubated in primary antibody overnight at 4°C. Following the third wash, coverslips were incubated in secondary antibody for 1h at RT. Coverslips were mounted on slides using ProLong Gold Antifade Reagent with DAPI (Cell Signaling Technology). Imaging immunofluorescence slides was done with Nikon W-1 spinning disk confocal microscope at UMB confocal core facility.

Cell viability assays: Crystal violet, MTT, and Annexin V/PI assay

The efficacy of cisplatin in the cell lines was determined using crystal violet assay kit (Abcam), 3-[4,5-dimethylthiazole-2-yl]-2,5-diphenyltetrazolium bromide (MTT) assay kit (Abcam) and Dead Cell Apoptosis kit with Annexin V FITC and Propidium iodide kit for flow cytometry (Invitrogen). All assays were performed according to the manufacturer's protocol. Briefly, FITC annexin V diluted 1:2 in annexin V-binding buffer and 10µg/mL PI solution were used. 200,000 cells were collected per condition and stained with 1µL FITC Annexin V and 2µL PI solution. Flow Cytometry was performed on BD Canto II at UMB Flow Cytometry Core. Absorbance measurements for crystal violet (570nm) and MTT assay (590nm) were done using BioTek Cytation 5.

Neutral single cell gel electrophoresis or comet assay

For the detection of double strand break DNA damage, the neutral version of the comet assay was performed according to R&D Systems specifications (4250-050-K). Briefly, 1000 HNSCC cells were added to 1% low melting point agarose at 37 °C and was spread on a slide. Gel was allowed to solidify at 4°C and the slides were immersed in lysis solution and kept at 4 °C overnight. The slides were incubated in a cold neutral electrophoresis solution (Tris Base and sodium acetate, adjusted to pH = 9.0 with glacial acetic acid) for 30 min to allow DNA unwinding. Electrophoresis was performed at 4°C, 27V, and 300 mA for 35 minutes. The slides were then immersed in DNA precipitation buffer (7.5M NH_4Ac, 95% EtOH), immersed in 70% EtOH for 30 minutes at RT, dried at 37°C for 15 minutes, and stained with SYBR green. A total of at least 30 cells were randomly selected per treatment and examined using a fluorescence microscope (Cytation5) at 200× magnification for image analysis using OpenComet software. To determine DNA damage, the percent tail intensity (also known as % tail DNA), tail length and olive moment (product of tail DNA% and the distance between the intensity-weighted centroids of head and tail) were used.

In vitro extrachromosomal homologous recombination repair efficiency assay

Extrachromosomal HR assay was performed according to (2). Nuclear extracts from HNSCC cells were collected using the Cell Lytic NuCLEAR Extraction Kit (Sigma Aldrich). Extracts were dialyzed for two hours using GE Healthcare Plus One Mini Dialysis Kit 1kDa in dialysis solution. Following protein content determination using Nanodrop, 2.5μg nuclear extracts were incubated with 5μL each of dl-1 and dl-2 plasmid (Norgen Biotek HR kit) in reaction buffer for 2 hours at 30°C. Plasmid DNA was isolated by QIAamp DNA mini kit (Qiagen), and quantitative real-time

PCR QuantStudio 3 (ThermoFisher Scientific) performed to determine the relative quantity of recombined plasmid products using primers (Norgen Biotek) that spanned the repair site, normalized against amplification of a distant site.

HNSCC tumor derived organoids generation and characterization

HNSCC tumor derived organoids generation, culture and genomic characterization was already described in (42). RNA sequencing was performed following the CEL-Seq protocol.

Tumor spheroid culture, imaging, and viability assay

For qualitative studies, (3D) tumor spheroids were created using low-attachment culture plates. Briefly, 3000 cells were plated in a 96-well round-bottom low attachment plate (BioFloat from Sarstedt) with 200µL of complete DMEM media, centrifuged at 300xg for 10 min, and incubated for 96 hours in a humidified incubator (5% CO2, 37 °C). Following successful spheroid formation, 100uL of media was removed per well. Drugs were added in 100µL of media for 24hours (5% CO2, 37 °C). The spheroids were stained with (50ug/mL) Propidium Iodide and imaged with Celigo Imaging Cytometer.

For quantitative studies, the RASTRUM Platform (Inventia Life Science, Australia; UMB Translational Shared Services Core) was used to create matrix-embedded 3D models (96-well plate) of our HNSCC cells according to (86). Matrices used for the PrintRun include Px02.28P, ~ 1.1kPa, containing GFOGER, RGD, DYIGSR. For the inert base, 1x 1500 µL Activator F3, 1x 1500 µL Bioink F32, 1x 200 µL Bioink F239, 1x 200 µL Activator F177 were used. After cells were harvested and counted, media containing 400,000 cells was transferred into a sterile microcentrifuge tube and centrifuged to pellet cells. Cell pellets were resuspended in the

biofunctional activators. Once the cartridge was inserted with the lid removed, model printing began. After printing, cells were maintained in complete DMEM (containing FBS and antibiotics) for at least 48 hours prior to drug treatment. Following drug treatment, spheroid viability was determined using the Cell Titer-Glo 2.0 Assay (Promega) by incubating spheroids in 100μL of media and 100μL of Cell Titer Glo solution for 30 minutes. Luminescence was measured with Cytation5.

ABC Transporters Assay, RNA extraction, and cDNA synthesis

Genes expression of human ABC transporters was profiled using the Human ABC Transporters TaqMan™ Array Fast 96-well (Thermo Fisher Scientific Cat# 4418811). The gene levels for the 44 human ABC transporters and four endogenous controls were analyzed by real-time PCR using the QuantStudio™ system with TaqMan™ Fast Advanced Master Mix (Thermo Fisher Scientific Cat# 4444557). 80ng of cDNA was used as a template per TaqMan reaction. Samples preparation and thermal cycling conditions were conducted according to the manufacturer's instructions. Total RNA was extracted from HN4 and HN4 A4 cells using RNeasy Mini Kit (Qiagen Cat# 74104). Reverse transcription (RT) was done using 2μg of total RNA per reaction and a High-Capacity cDNA Reverse Transcription Kit with RNase Inhibitor according to the manufacturer's instructions (Fisher Scientific Cat# 4374966). The expression level of each gene was analyzed in triplicate. Raw Cq-values obtained were used to calculate the fold change ($2^{-\Delta\Delta C_t}$).

Subcellular Protein Fractionation

Subcellular protein fractionation was performed using kit from ThermoFisher Scientific and performed according to kit protocol. Extraction buffers were prepared, and 1 million cells were

used per cell line. Following extractions, subcellular fractions were prepared for Westerns using DTT and SDS-PAGE Sample Loading Buffer (G Biosciences). Western blot analysis and immunoprecipitation were performed as in (8). For transfer of proteins to PVDF membrane Bio-Rad Trans-Blot Turbo transfer system and protocol was used.

Site-directed mutagenesis

Site-directed mutagenesis was performed using the site-directed Quick Change II XL Site-Directed Mutagenesis Kit from Agilent Technologies (#200521). Tyr 54 and Tyr 315 residues of RAD51 were mutated to phenylalanine. CMV-hRAD51 plasmid containing RAD51 insert (125570) was used as the template, and the mutagenic forward and reverse primers were used for PCR. The details of the primer sequences are provided below. The PCR-amplified products were digested with Dpn I restriction enzyme that digests the parental, non-mutated DNA. The Dpn I-treated DNA was then transformed into the XL10-Gold ultracompetent cells per the manufacturer's instructions. The generated plasmids were sequenced to confirm the mutations at the desired position.

List of primers used for Site-Directed Mutagenesis

S. No.	Amplicon	Primer sequence (5'------------3')
**1**	RAD51-Tyr54	Forward- AAGCGGTCGCATTCGCACCGAAAAAGAGC<br>Reverse- GCTCTTTTTCGGTGCGAATGCGACCGCTT
**2**	RAD51-Tyr315	Forward- CACGCATCTGCAAAATCTTTGACTCCCCATGCC<br>Reverse- GGCATGGGGAGTCAAAGATTTTGCAGATGCGTG

Sex as a biological variable

Our study examined female mice because female animals exhibit less variability in phenotype and lesser aggression.

Mouse xenografts assays

Athymic (nu/nu) 8-week-old female nude mice were supplied by Jackson (Jax®) Laboratories. All animal experiments were carried out after review and approval by the Johns Hopkins Institutional Animal Care and Use Committee and animals were treated according to standard international guidelines. Briefly, CAL27 and CAL27 A4 KO cells were resuspended in DMEM (serum-free medium) containing 30% matrigel before 100 µl (2 million cells) was injected subcutaneously in the right and left flanks of athymic nude mice using a 26.5-guage needle. Once the subcutaneous tumors were detected or reached a mean volume of 150 mm^3, mice were randomized into 4 groups (for cisplatin sensitivity experiments) and 8 groups (for drug combination experiments) with 5 mice per group and began treatment. The treatments were administered intraperitoneally at the following doses: (1) 5 mg/kg cisplatin, (2) 2 mg/kg EG00229, (3) 10 mg/kg EG00229, (4) 60 mg/kg Imatinib, (5) 5 mg/kg cisplatin and 2 mg/kg EG00229, (6) 5 mg/kg cisplatin and 10 mg/kg EG00229, (7) 5 mg/kg cisplatin and 60 mg/kg Imatinib, (8) PBS or control. All treatments excluding imatinib were administered three times per week. Imatinib intraperitoneal injections were administered five times per week. For the drug combination experiment, experiment was terminated 10 days after the start of the treatment, as the mice had lost 20 % of their initial weight. 24 hrs after the last administration of treatments, the mice were sacrificed and the tumors collected. Tumor volume was reported as $Volume = \frac{Length*Width^2}{2}$. Microvessel density was determined as

in (21), using CD31 immunohistochemical staining with CD31 antibody from Abcam, according to the manufacturer recommendations.

Study approval

All animal experiments were performed in accordance with the Animal Care and Use Program at Johns Hopkins University and were approved by the Institutional Animal Care and Use Committee (IACUC). HNSCC patient tumor-derived organoids (PTDOs) had been previously generated and described (42).

Statistics

Statistical analyses were conducted using the GraphPad Prism 10.0 statistical software. Data were expressed as mean ± standard error of the mean (SEM) from at least 3 independent experiments. To determine statistically significant differences between test groups, unpaired student's t-test, one-way and two-way analysis of variance (ANOVA) with follow-up Bonferroni post hoc test. t-test was used for analyzing differences between two groups and one- and two-way ANOVA for more than two groups followed by post-hoc analysis. Normalization was done using CTRL non-treated group; for Westerns, protein band intensities were normalized to loading control (GAPDH and Vinculin) and relative to CTRL band intensities. ns $p>0.05$, *$p<0.05$, **$p<0.01$, ***$p<0.001$, and ****$p<0.0001$.

List of antibodies

Rabbit anti-human ANGPTL4	PTG	18374-1-AP; RRID: AB_2878539
Mouse anti-human c-ABL (8E9)	Santa Cruz Biotechnology	sc-56887; RRID: AB_781732
Recombinant anti-neuropilin 1 [EPR3113]	Abcam	ab81321
Recombinant anti-Rad51 antibody [EPR4030(3)]	Abcam	ab133534
Anti-phospho-Histone H2A.X (Ser139) antibody	EMD Millipore	05-636-I
Rabbit Anti-human Histone H3 antibody	Cell Signaling Technology	CST9715S
Rabbit Anti-human HDAC2 antibody	Cell Signaling Technology	CST2540
Rabbit Anti-human GAPDH (D16H11) XP®	Cell Signaling Technology	CST5174
Mouse anti-human beta actin, clone 7D2C10	PTG	60008-1-Ig
Anti-Rabbit IgG (H+L) Antibody, Human Serum Adsorbed and Peroxidase-Labeled	KPL	4741516
Anti-Mouse IgG (H+L) Antibody, Human Serum Adsorbed and Peroxidase-Labeled	KPL	4741806
Horse anti-rabbit IgG antibody DyLight™ 594	VectorLabs	DI-1094-1.5
Horse anti-mouse IgG antibody DyLight™ 488	VectorLabs	DI-2488-1.5
CTR1/SLC31A1 Antibody	CST	13086S
Rabbit anti-CD31 antibody [RM1006]	Abcam	ab281583
Vinculin (E1E9V) XP® Rabbit mAb	CST	13901S

Figure 1G-J: ANGPTL4 Expression in Cell Lines

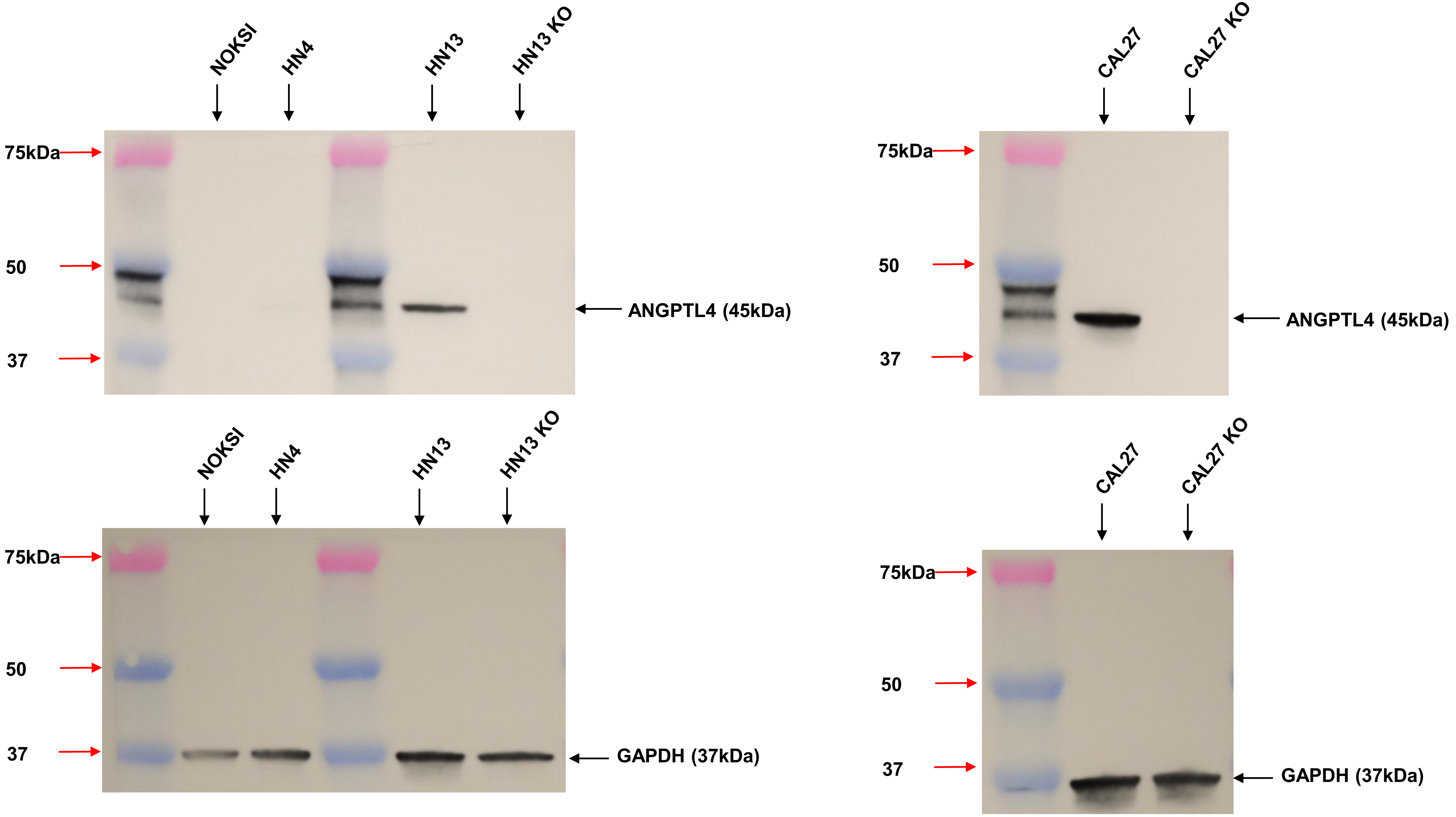

ANGPTL4 loss increases DNA damage in HNSCC cells in response to cisplatin

Figure 2I

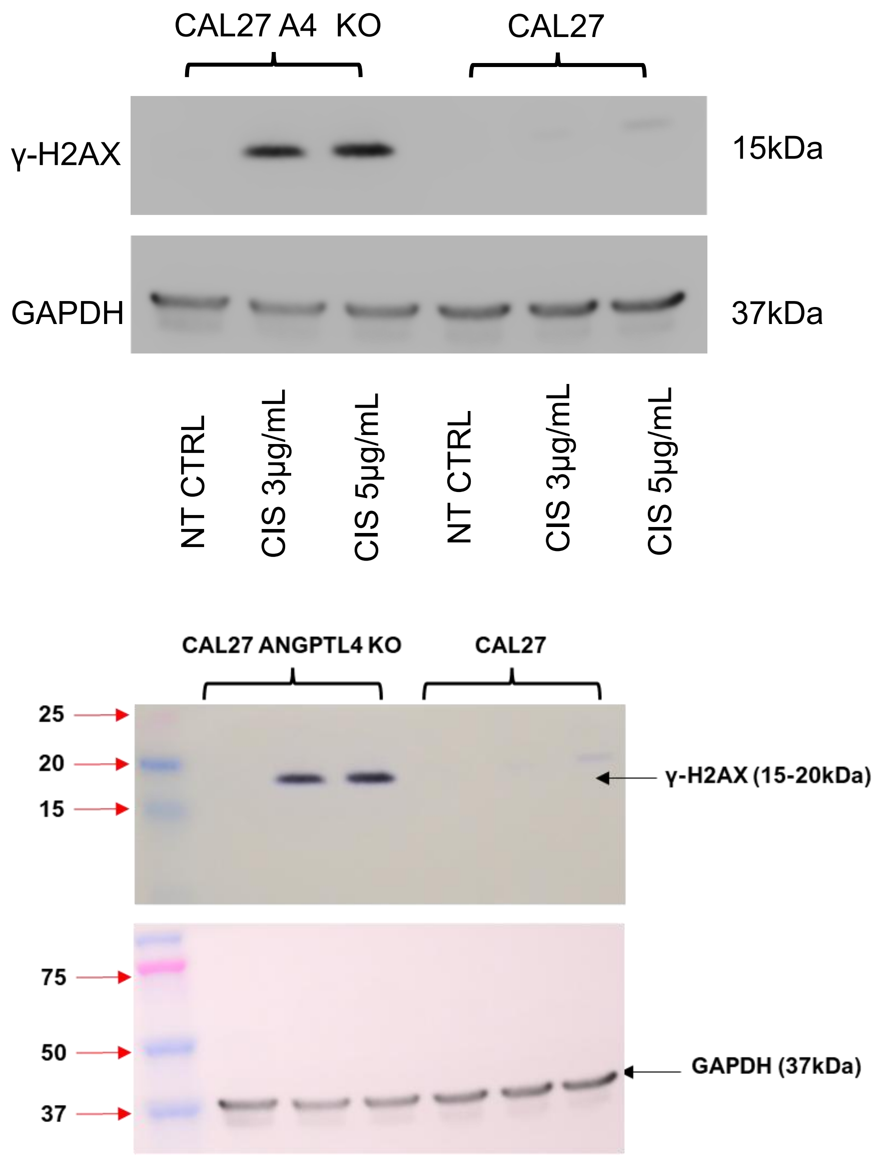

Figure 2K

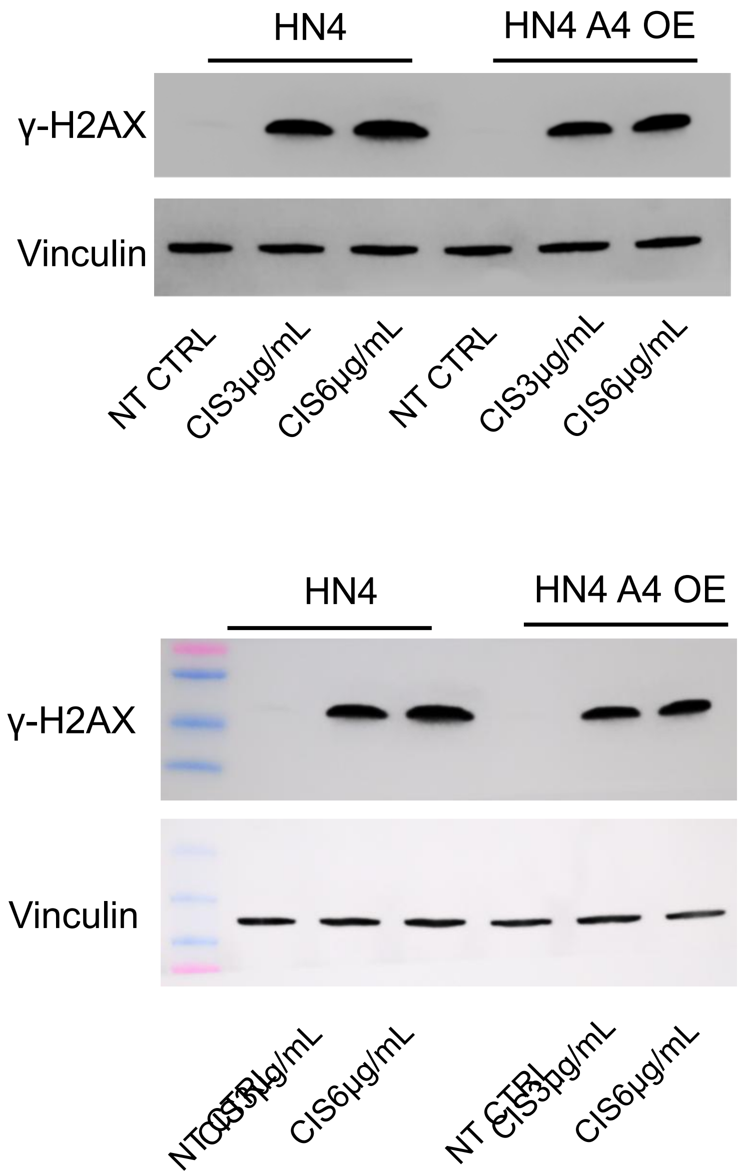

Figure 3A: Western blot analysis of RAD51 Y315 phosphorylation upon treatment of NOKSI with rhANGPTL4 full-length (A4 FL), for 5, 15, 30, and 60 minutes

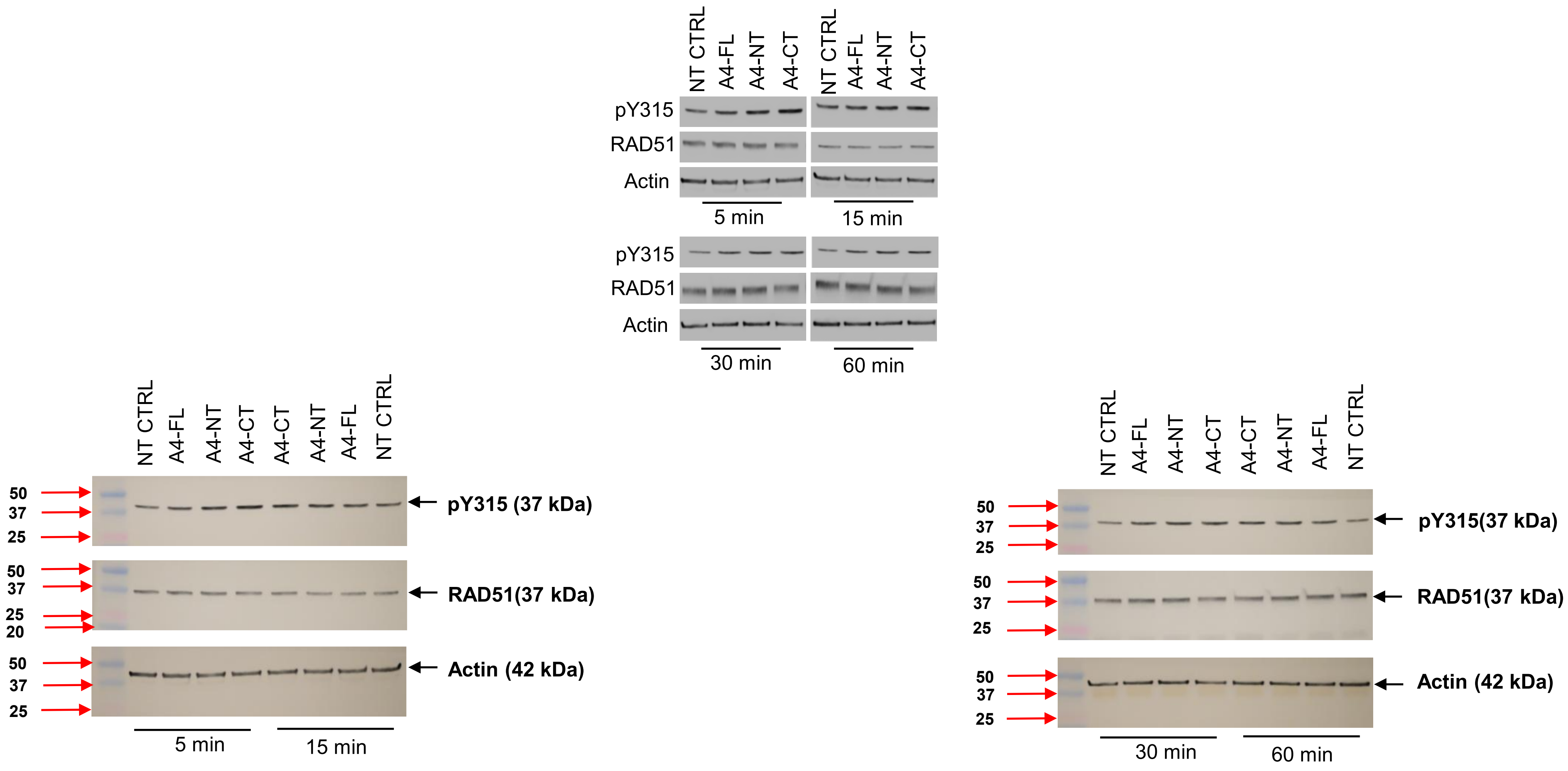

Figure 3C: Western blot analysis of RAD51 Y54 phosphorylation upon treatment of NOKSI with rhANGPTL4 full-length (A4 FL), for 5, 15, 30, and 60 minutes

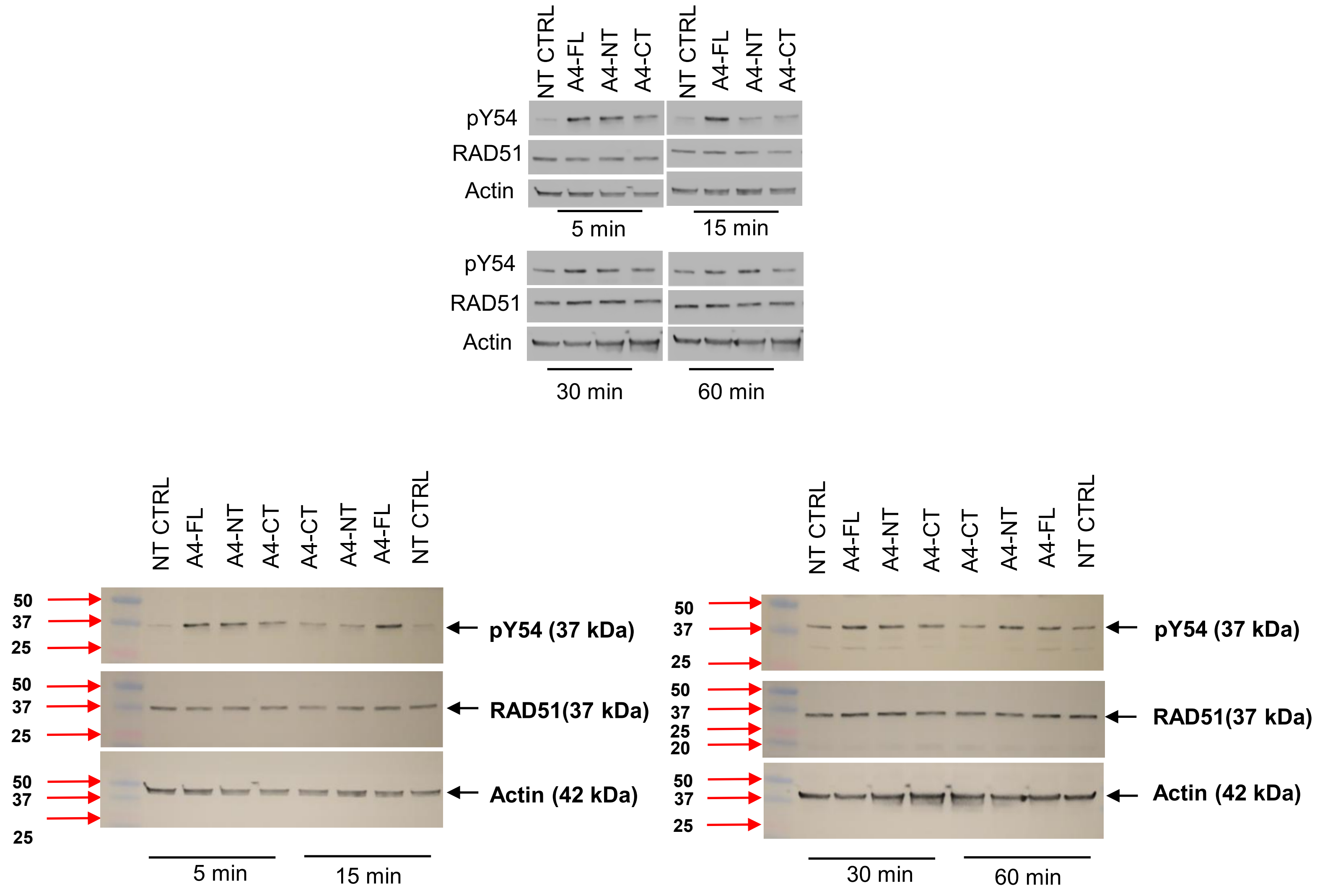

Figure 3E, F: NRP1 siRNA on RAD51 Phosphorylation in NOKSI treated with recombinant ANGPTL4

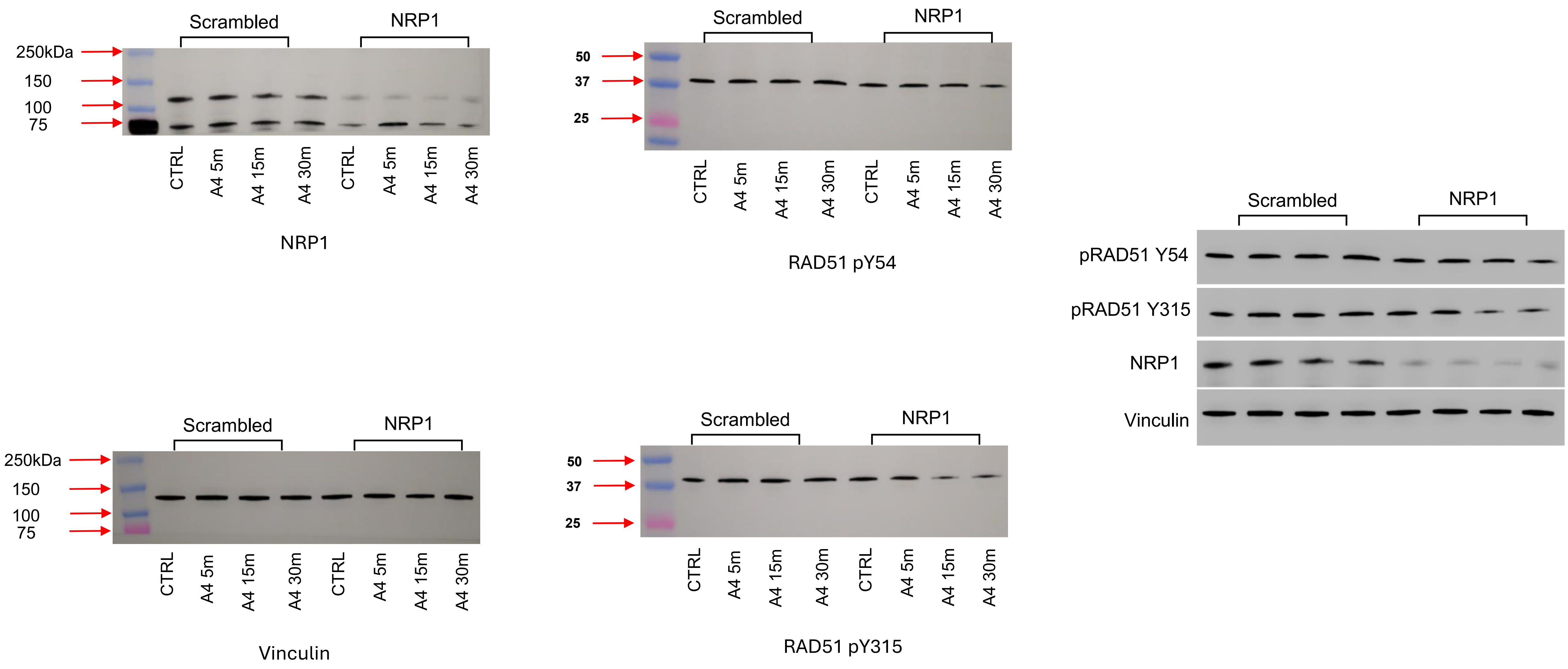

Figure 3I: ANGPTL4, RAD51, and ABL1 Fractionation (other data)

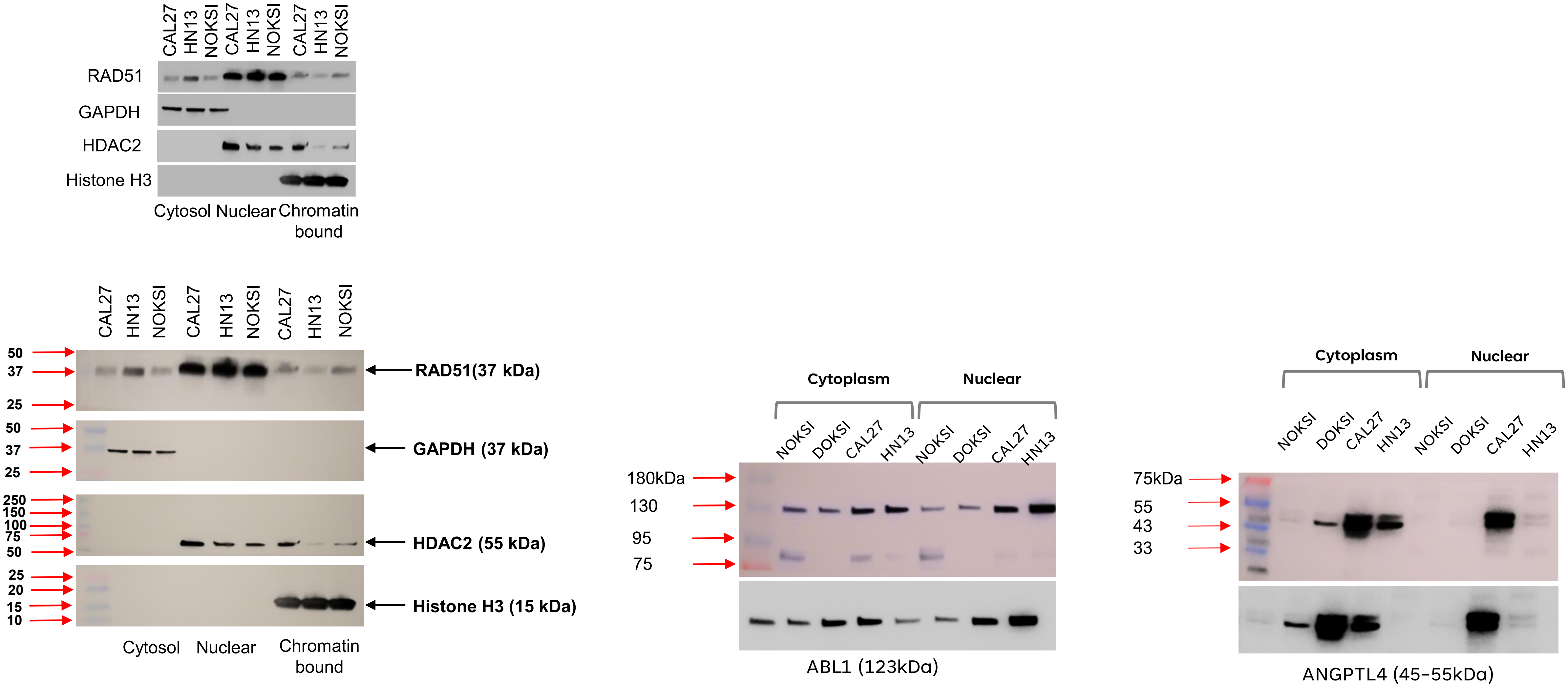

Figure 4I: NRP1 Inhibition (EG01377) impact on cisplatin sensitivity in CAL27 cells

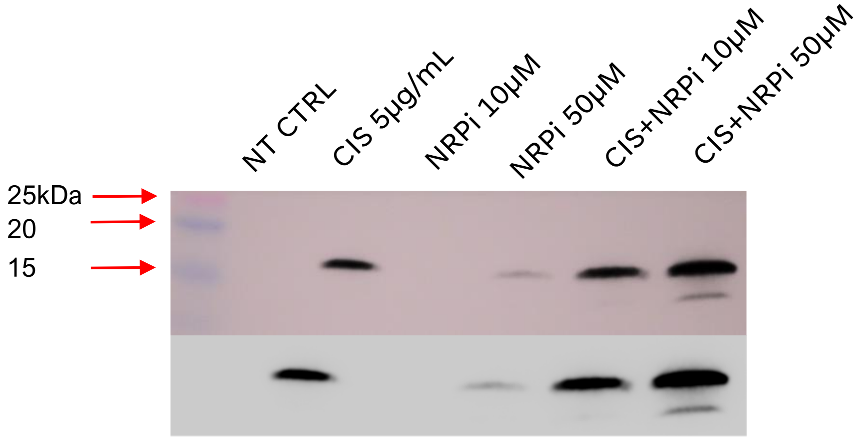

y-H2AX (15-20kDa)

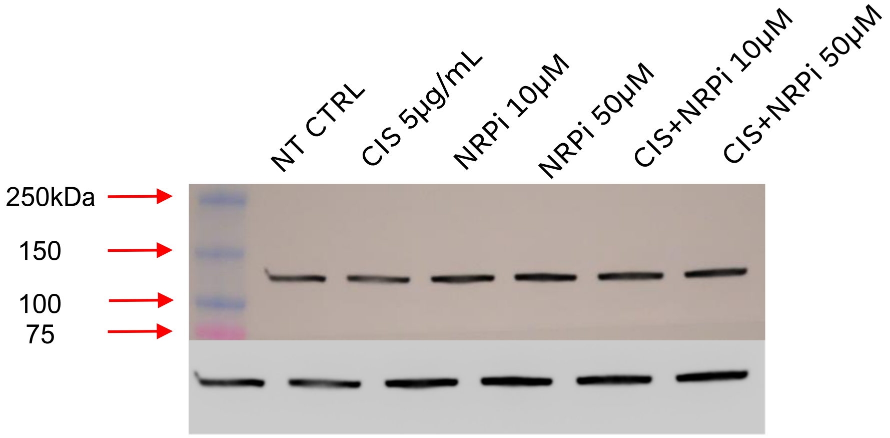

Vinculin (120kDa)

Figure 4K: NRP1 Inhibition (EG00229) impact on Extent of DNA Damage in CAL27 cells

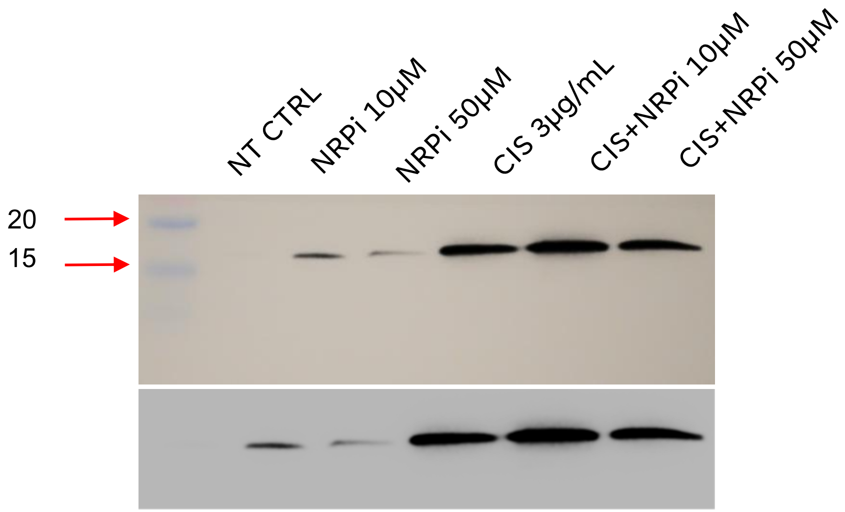

y-H2AX (15-20kDa)

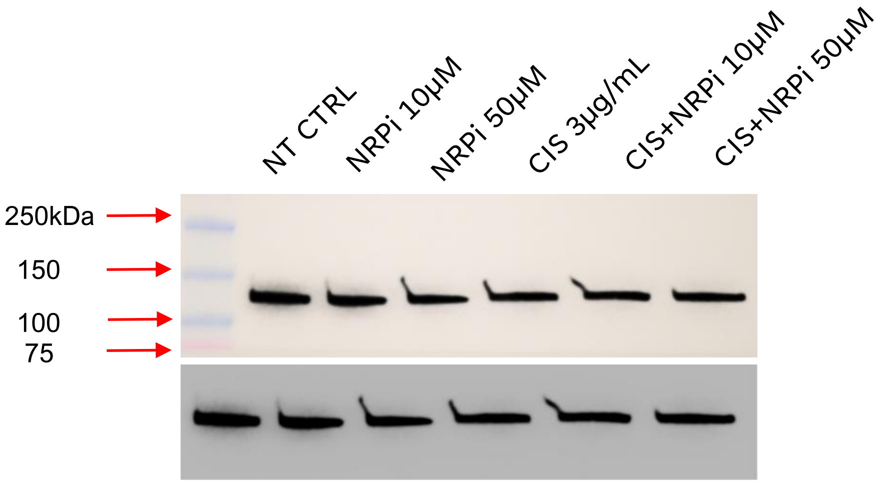

Vinculin (120kDa)

Figure 4M,N: Western blot analysis of RAD51 Y315 and Y54 phosphorylation upon treatment of NOKSI with rhANGPTL4 full-length (A4 FL) and NRP1 inhibitor (EG00229) 10μM and 100μM

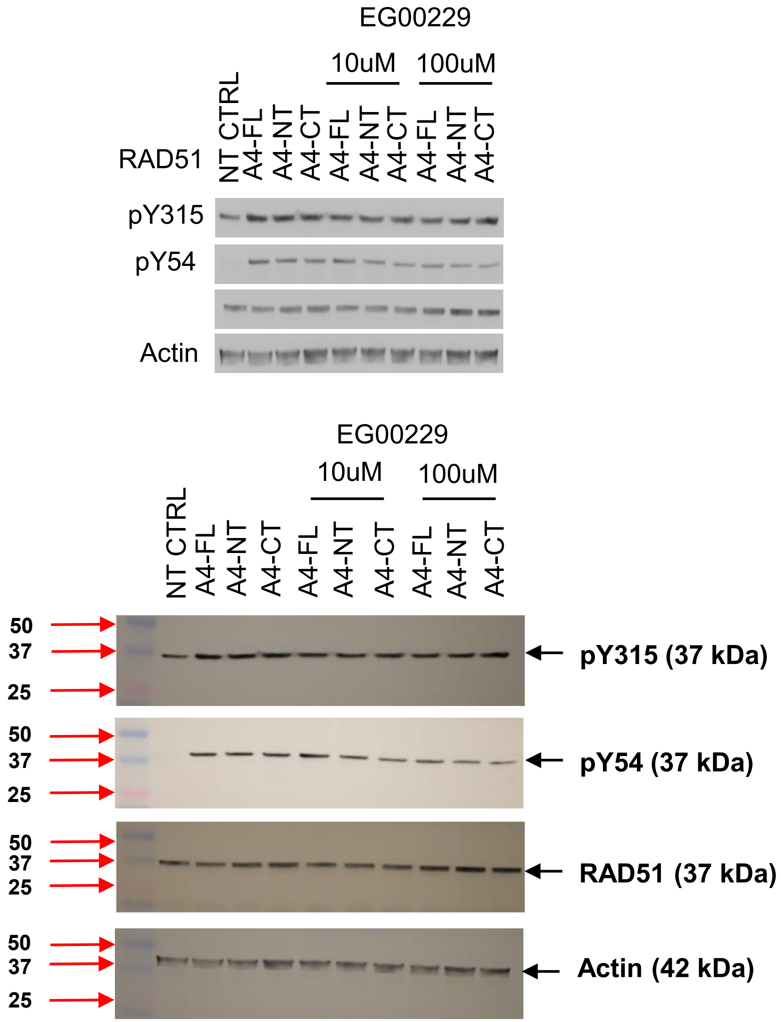

Figure 5I: Cisplatin and Imatinib (ABL1 inhibitor) in CAL27 cells

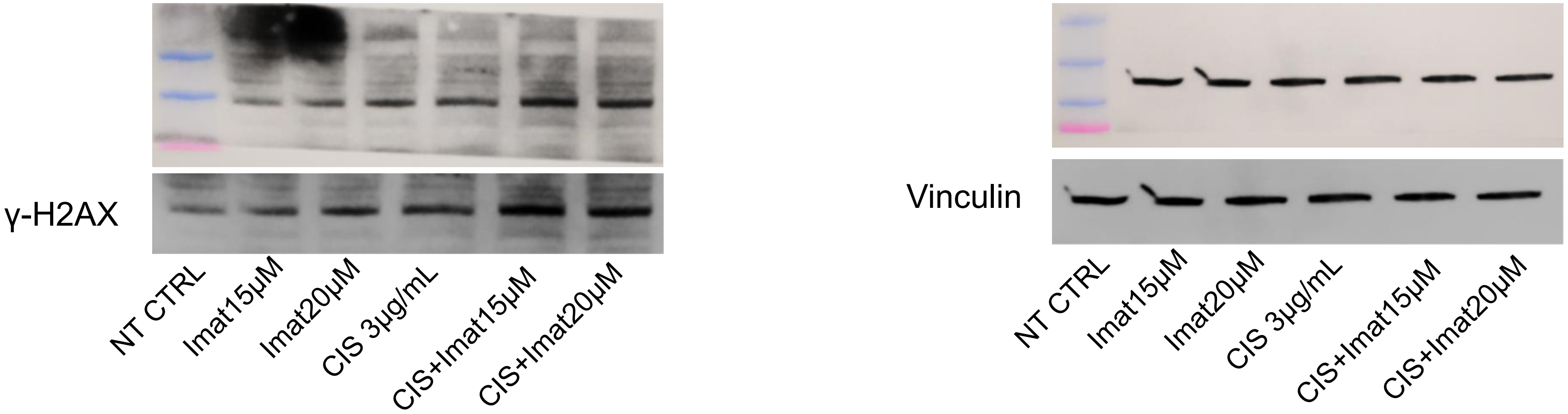

Figure 5K: Cisplatin and Dasatinib (ABL1 inhibitor) in CAL27 cells

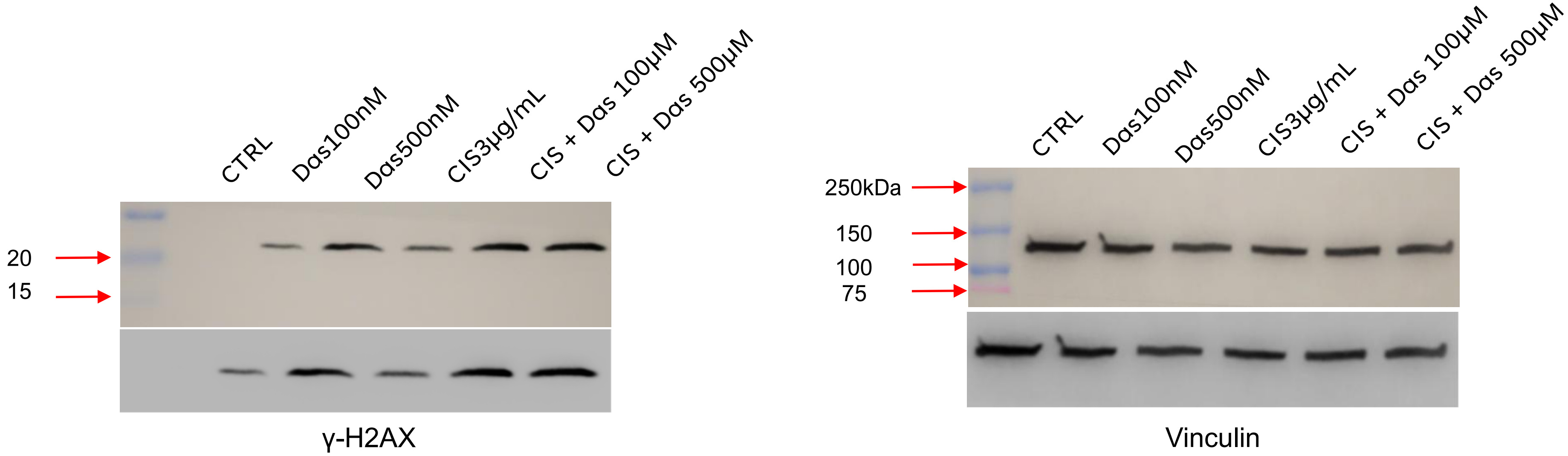

Figure 5M: Cisplatin and Dasatinib (ABL1 inhibitor) in HN13 cells

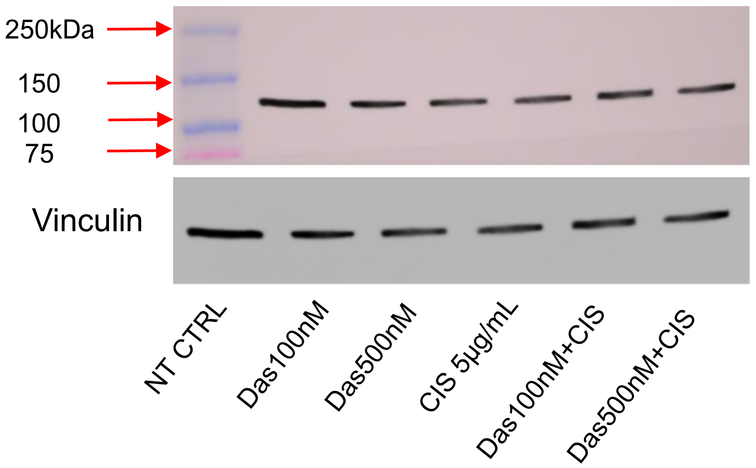

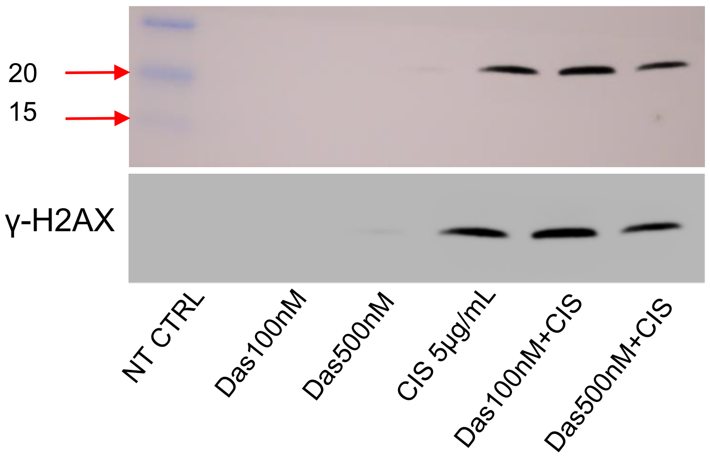

Figure 5O,P: Western blot analysis of RAD51 Y315 and Y54 phosphorylation upon treatment of NOKSI with rhANGPTL4 full-length (A4 FL) and knockdown of ABL1

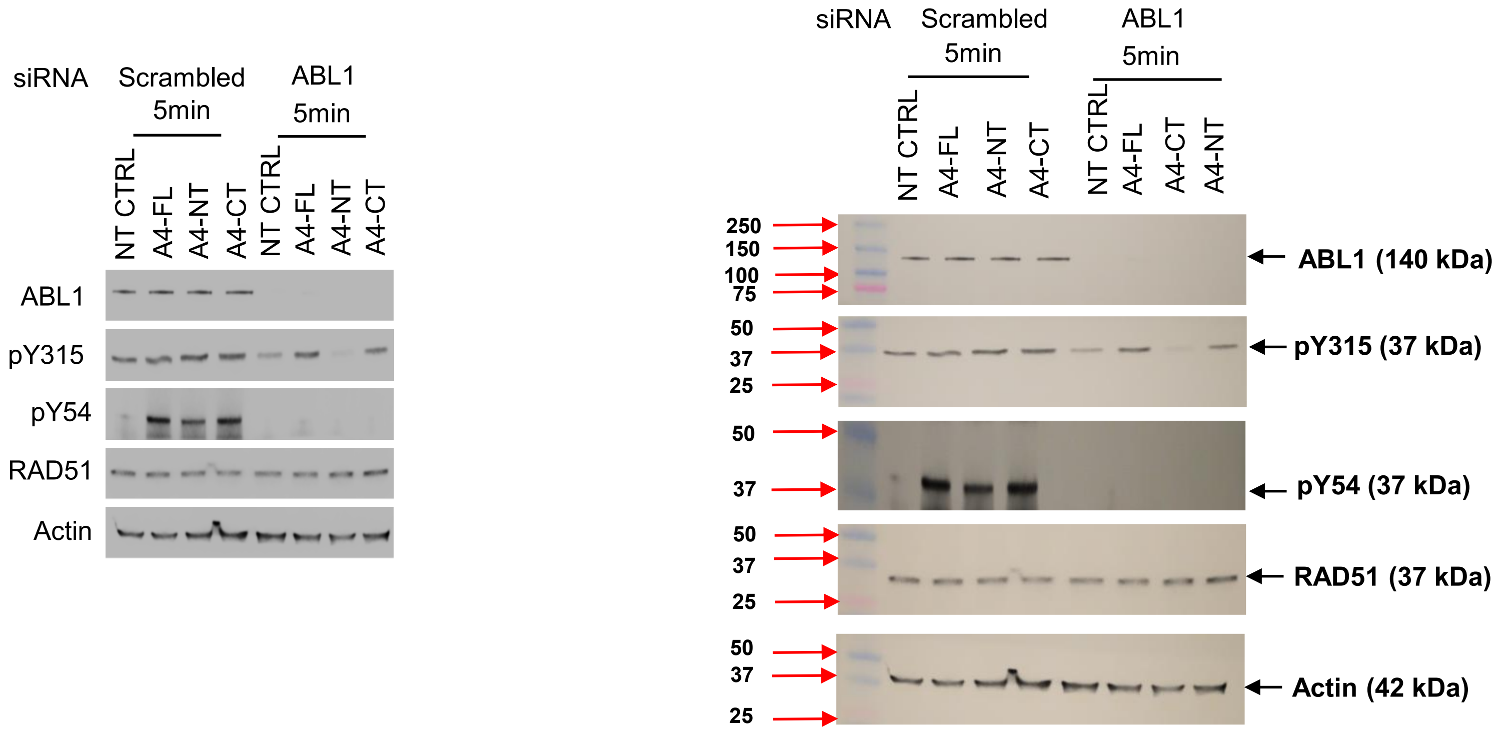

Supplementary Figure 1A: RAD51 Expression following HN4 and HN4 A4 OE treatment with Cisplatin

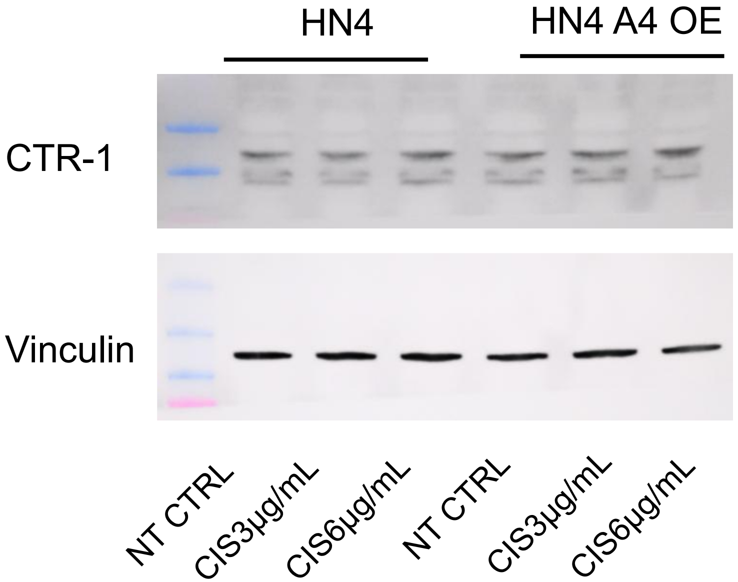

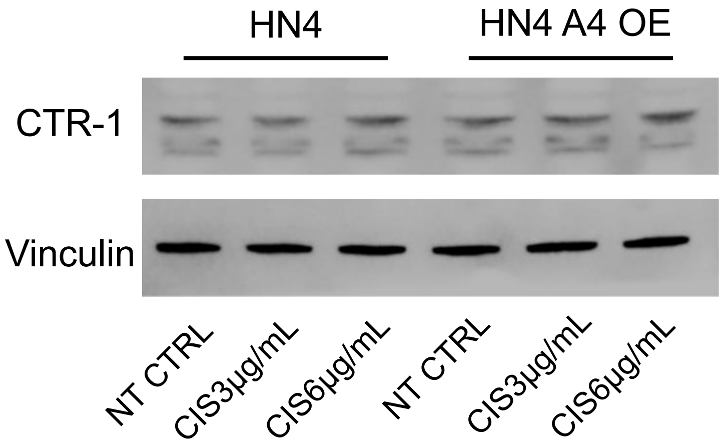

Supplementary Figure 2A: ANGPTL4 loss increases DNA damage in HNSCC cells in response to cisplatin

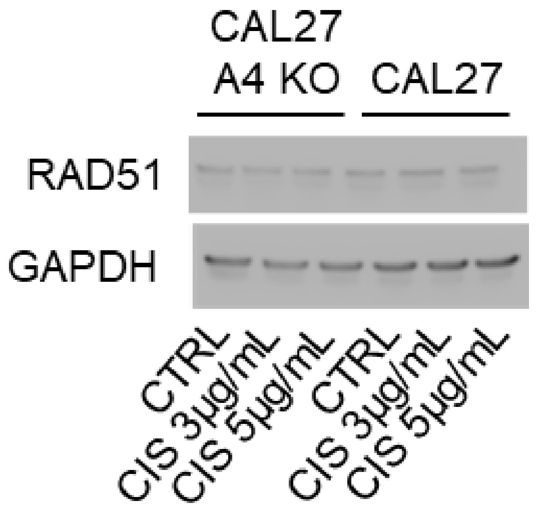

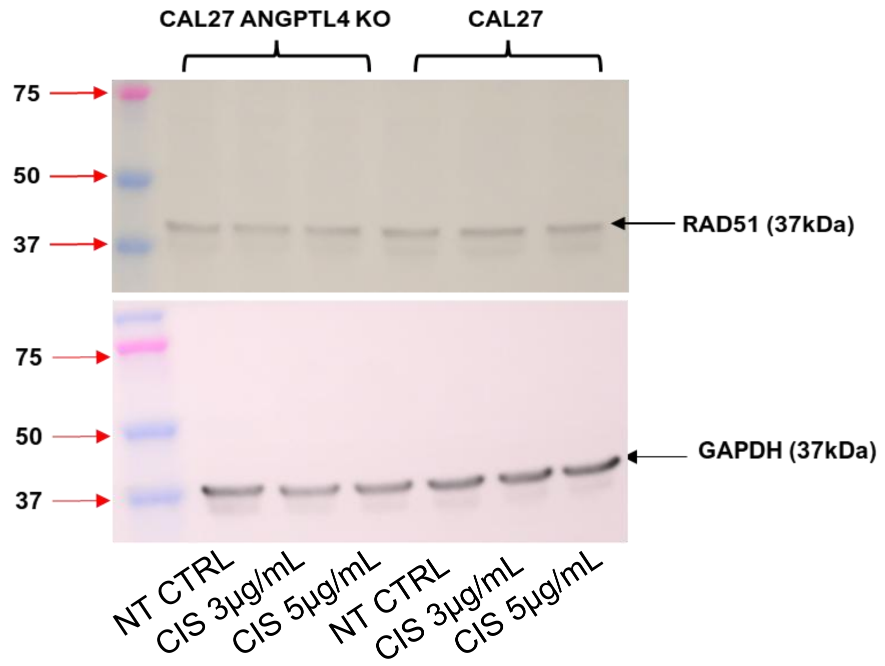

Supplementary Figure 3E: NRP-1 Inhibition (EG00229) impact on DNA damage response in HN13 cells

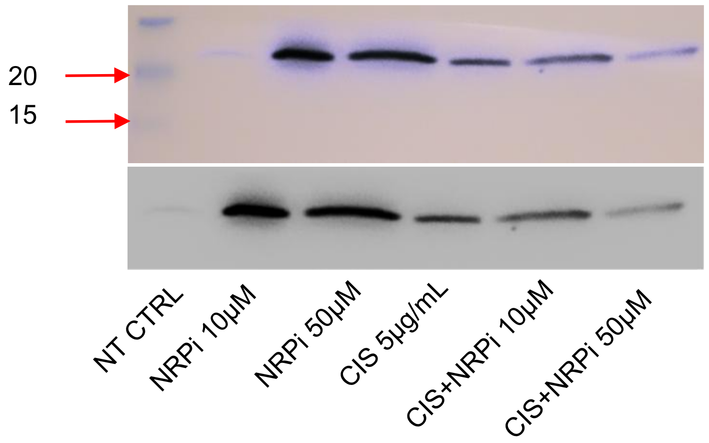

γ-H2AX

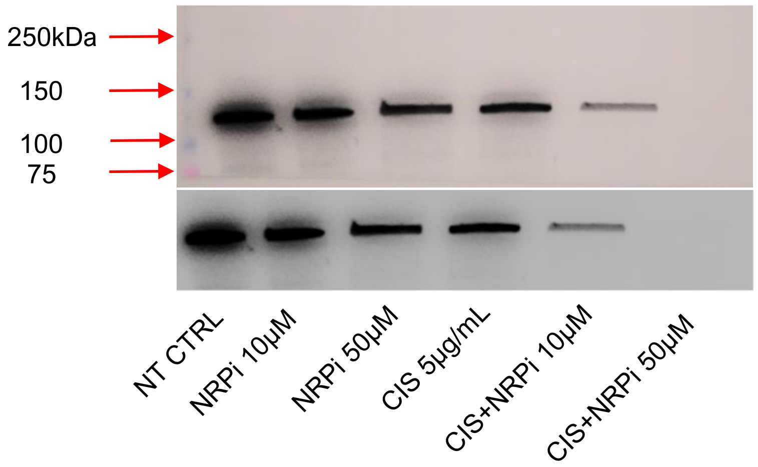

Vinculin
