# Supplementary material for: Targeting the ANGPTL4/NRP1/ABL1/RAD51 axis reverses cisplatin resistance by impairing DNA damage repair in head and neck cancer
Source: Proc Natl Acad Sci U S A. 2026 Mar 26;123(13):e2510265123. doi: 10.1073/pnas.2510265123 (PMC13038062; doi:10.1073/pnas.2510265123)

Figure 1G-J: ANGPTL4 Expression in Cell Lines

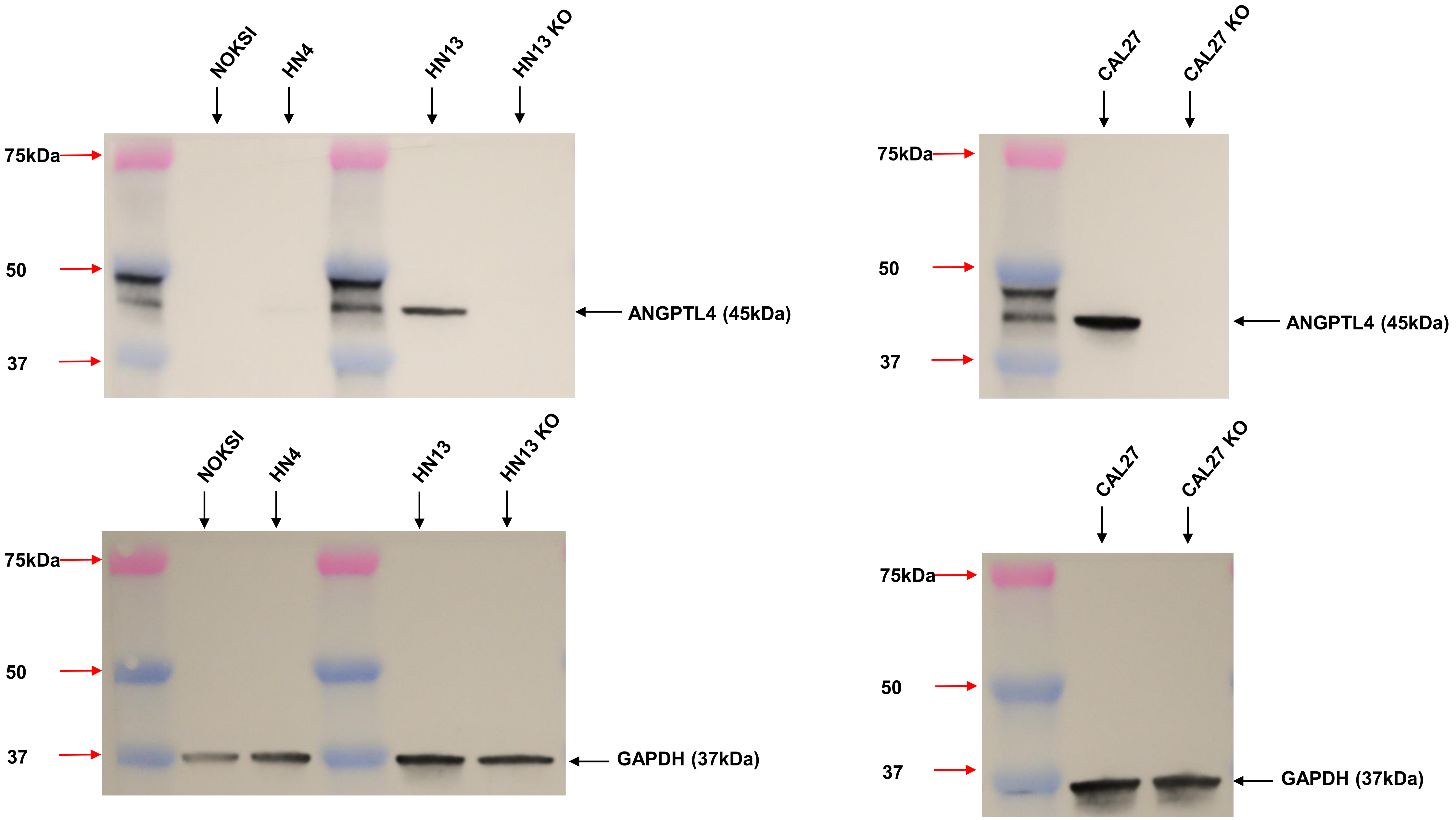

ANGPTL4 loss increases DNA damage in HNSCC cells in response to cisplatin

Figure 2I

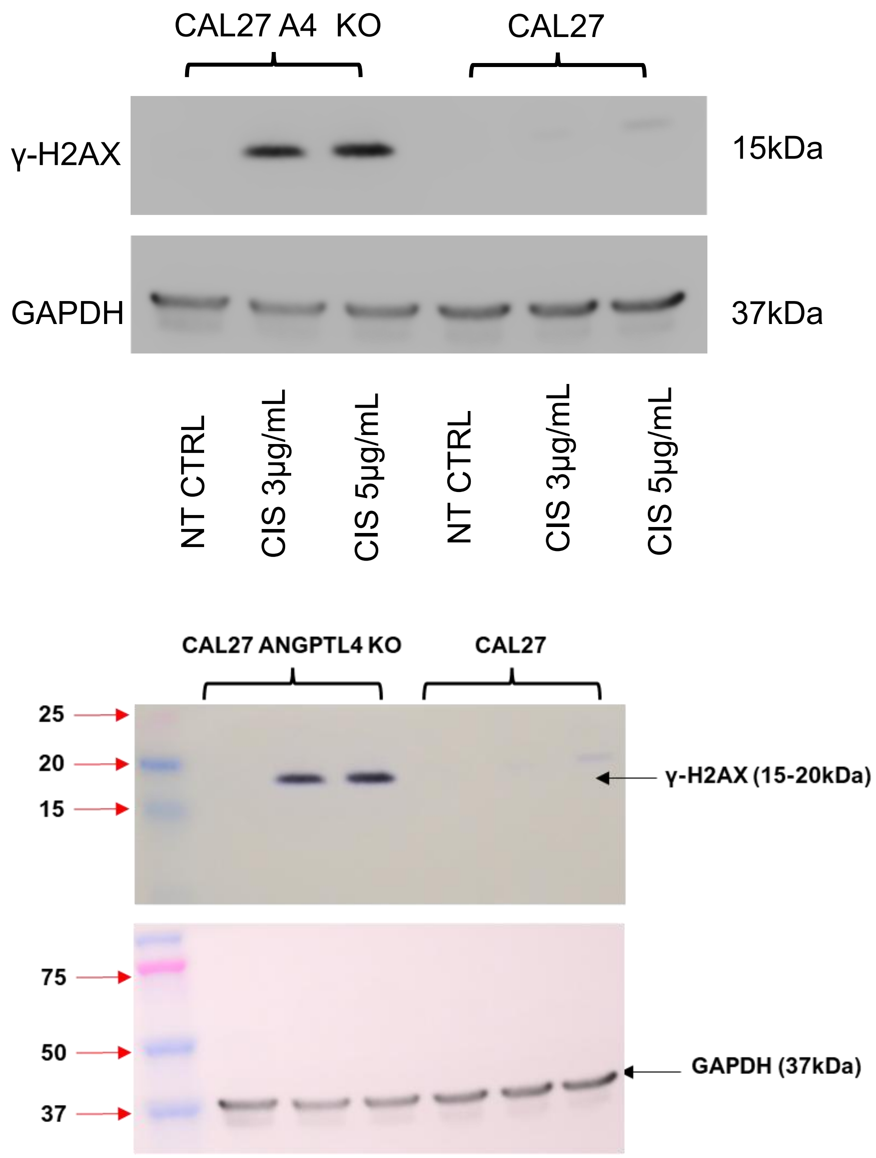

Figure 2K

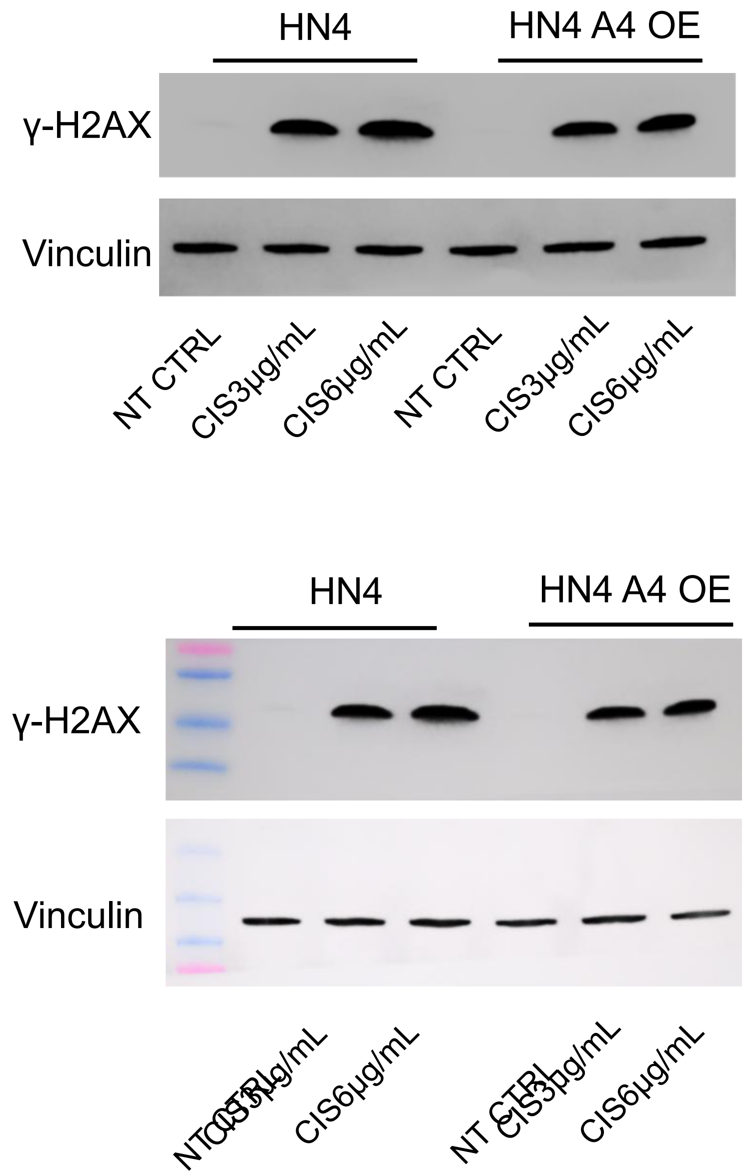

Figure 3A: Western blot analysis of RAD51 Y315 phosphorylation upon treatment of NOKSI with rhANGPTL4 full-length (A4 FL), for 5, 15, 30, and 60 minutes

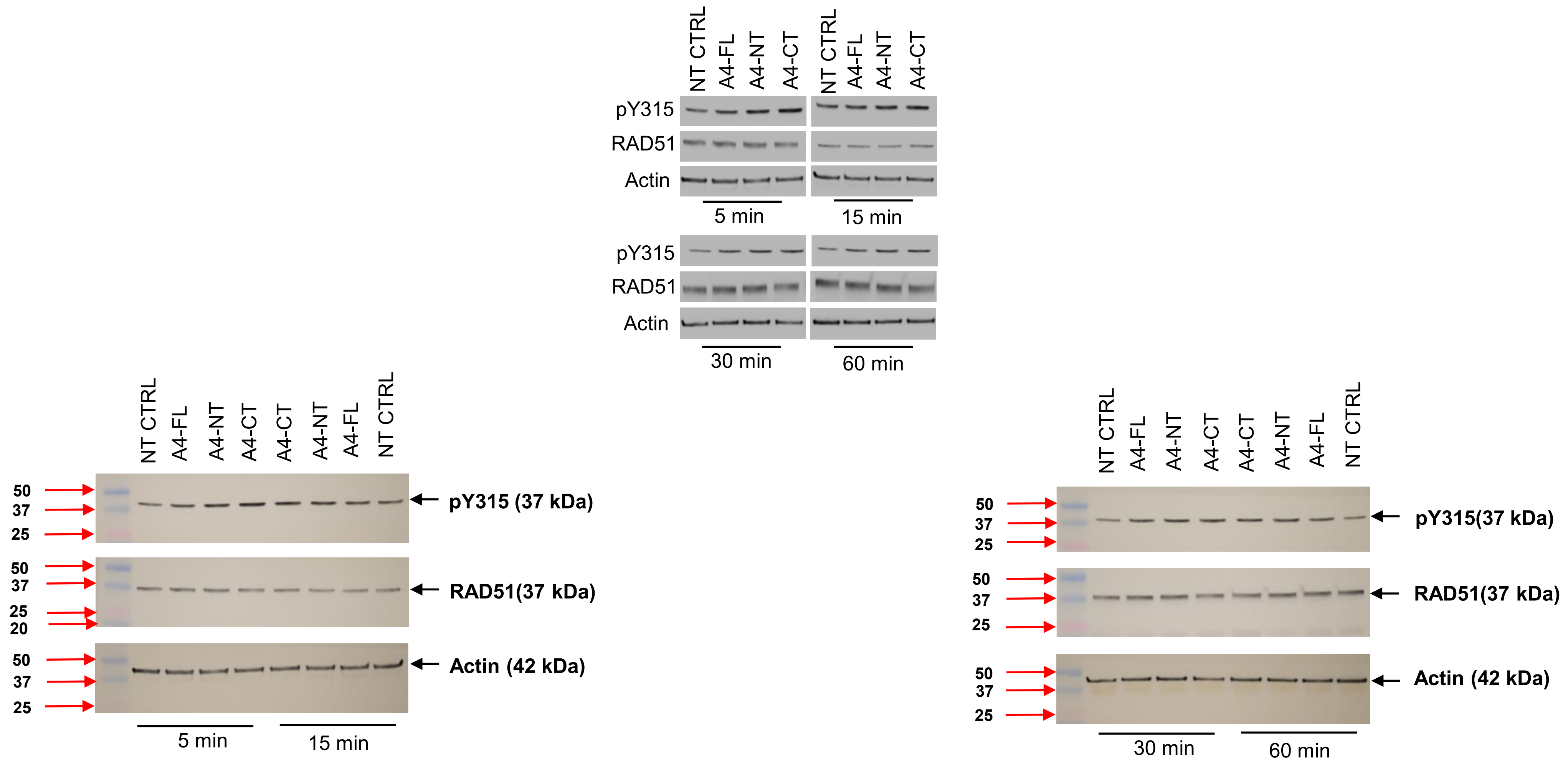

Figure 3C: Western blot analysis of RAD51 Y54 phosphorylation upon treatment of NOKSI with rhANGPTL4 full-length (A4 FL), for 5, 15, 30, and 60 minutes

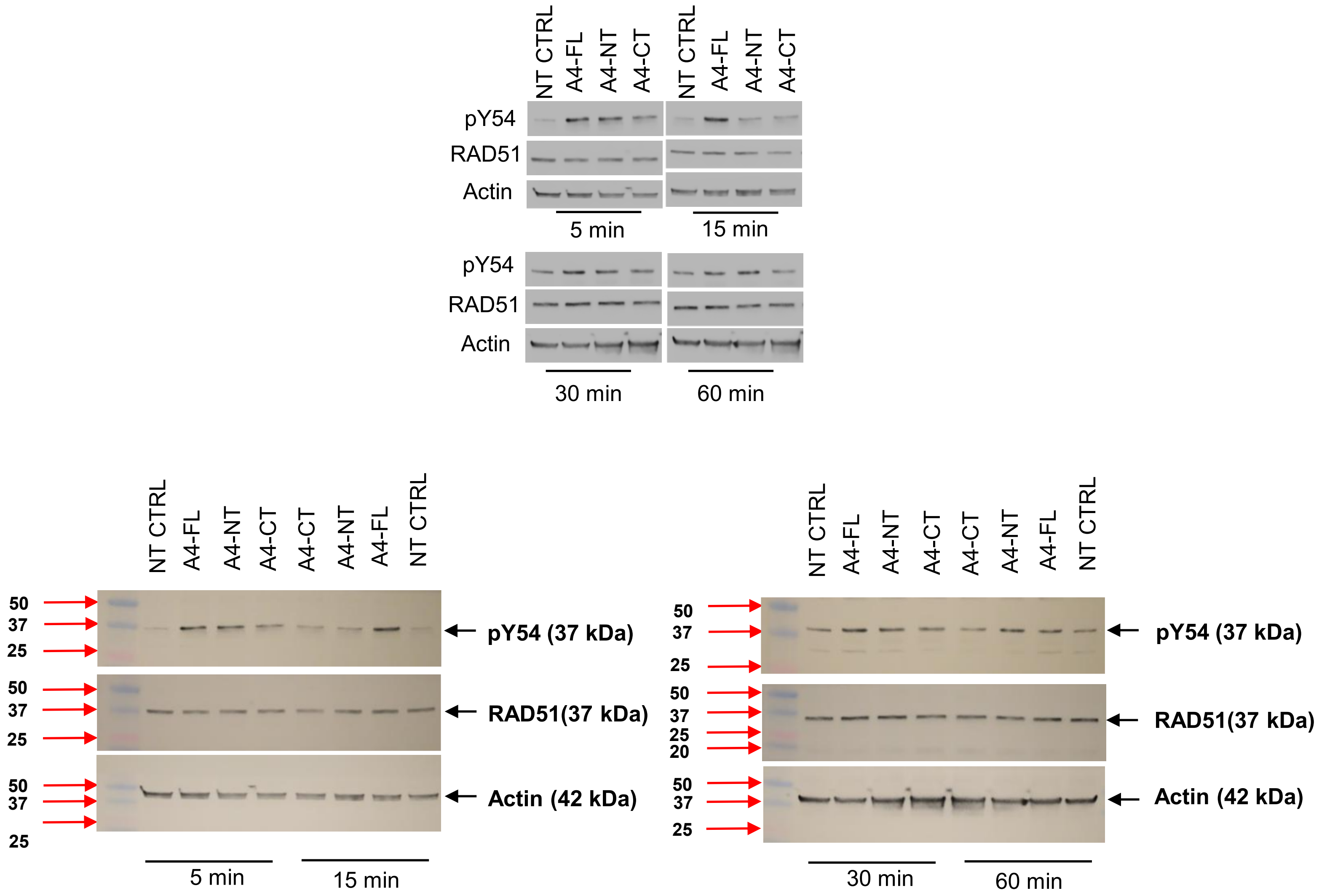

Figure 3E, F: NRP1 siRNA on RAD51 Phosphorylation in NOKSI treated with recombinant ANGPTL4

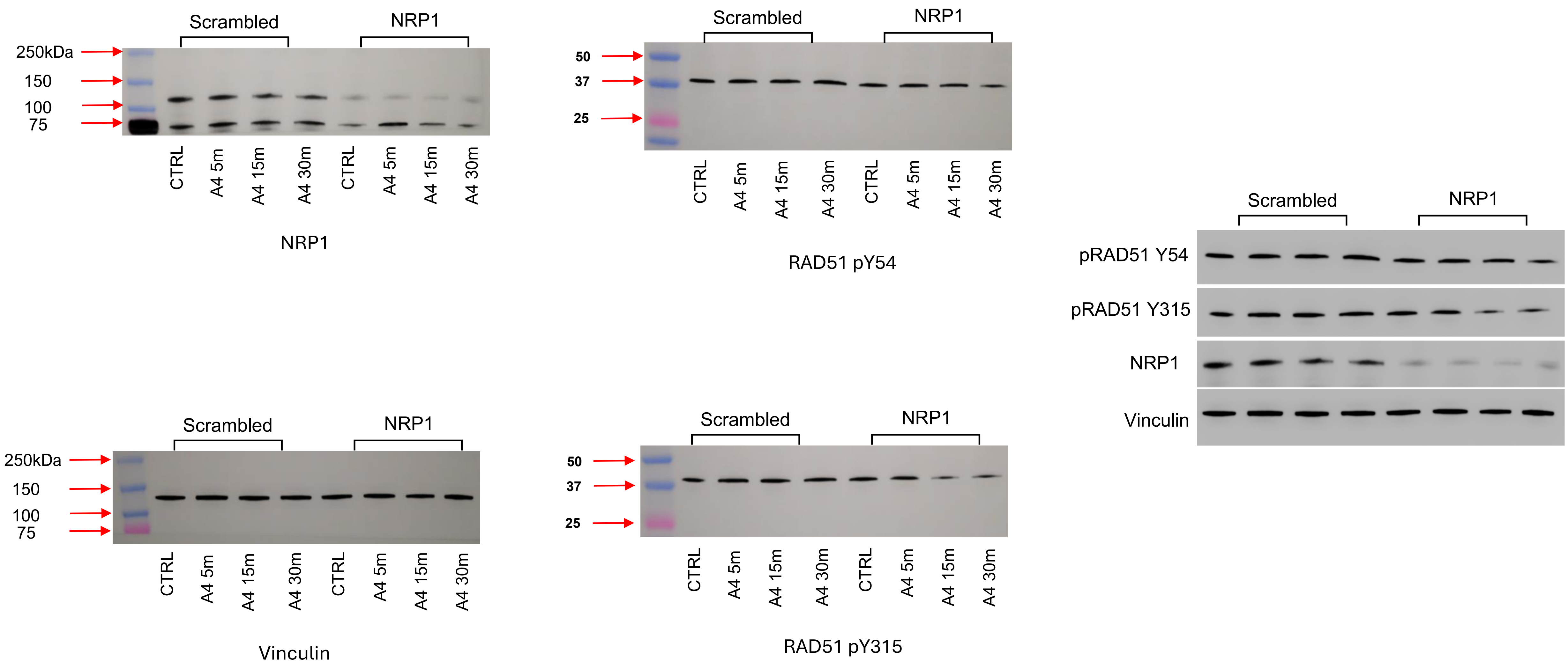

Figure 3I: ANGPTL4, RAD51, and ABL1 Fractionation (other data)

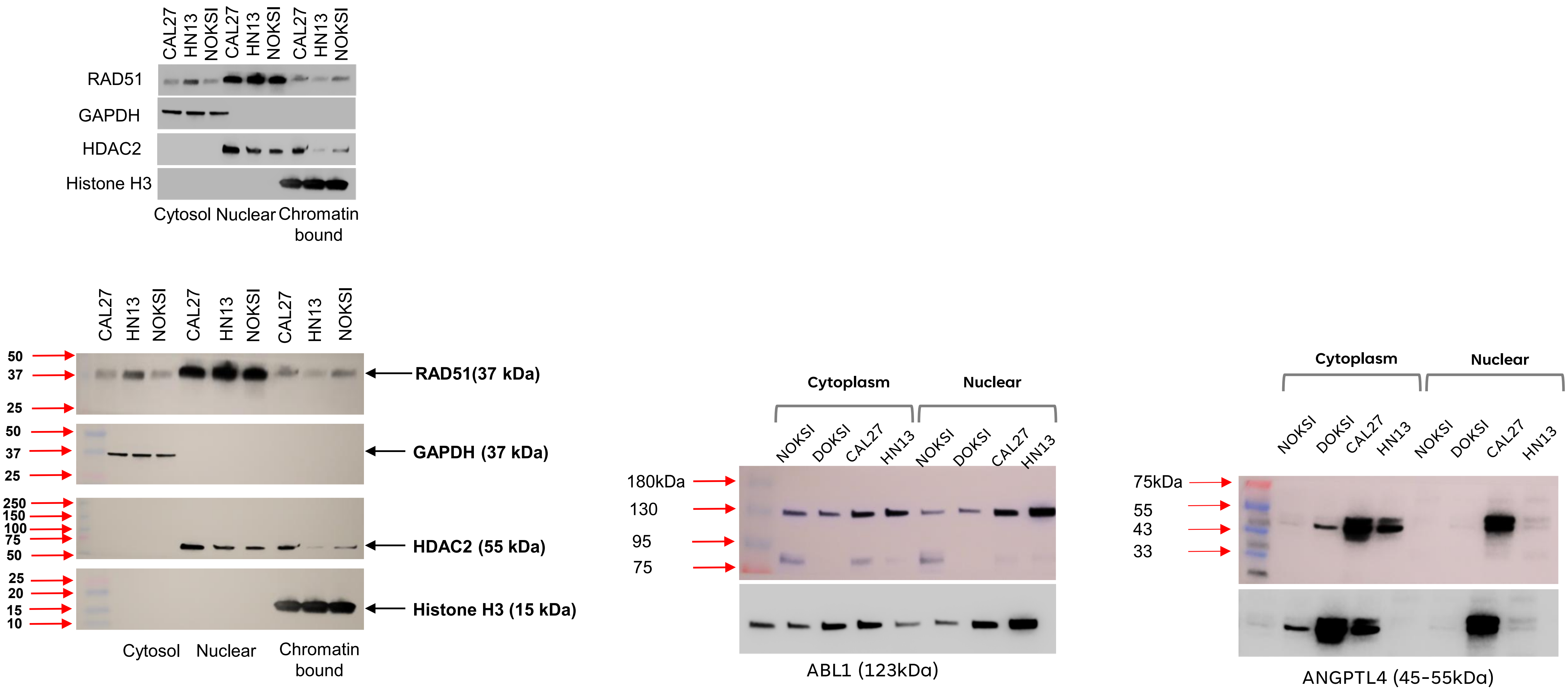

Figure 4I: NRP1 Inhibition (EG01377) impact on cisplatin sensitivity in CAL27 cells

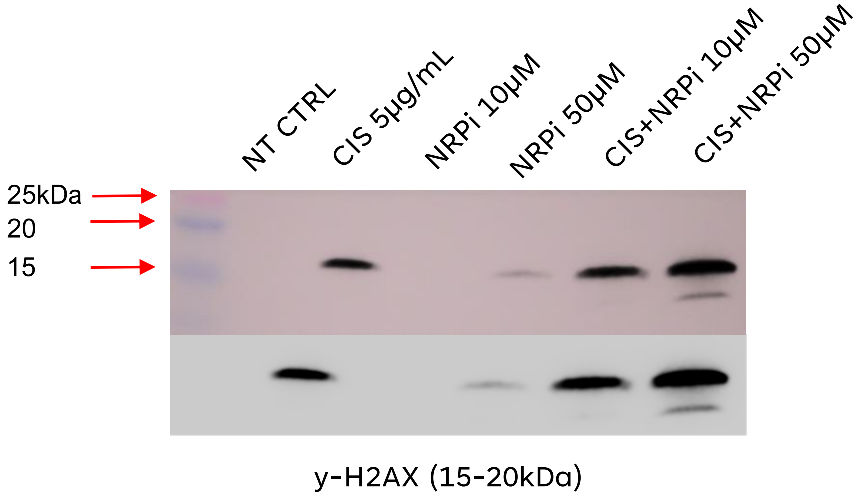

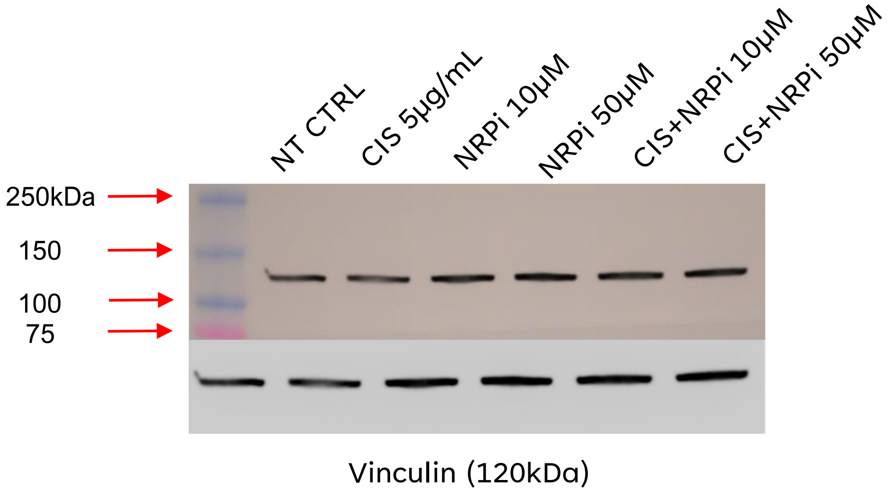

Figure 4K: NRP1 Inhibition (EG00229) impact on Extent of DNA Damage in CAL27 cells

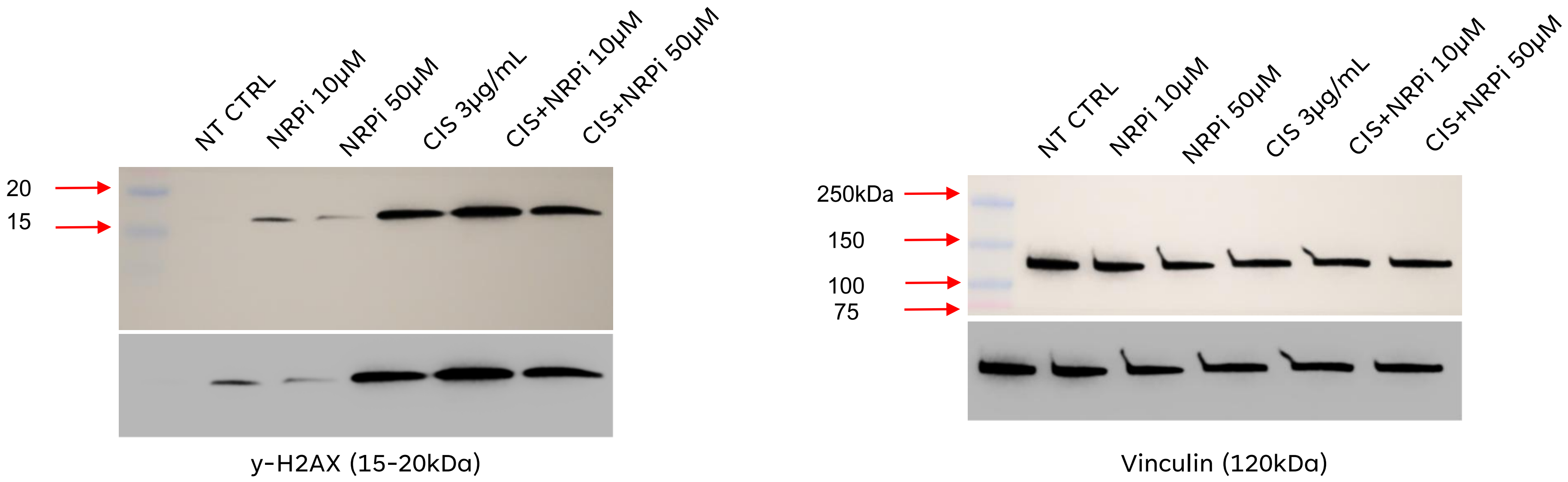

Figure 4M,N: Western blot analysis of RAD51 Y315 and Y54 phosphorylation upon treatment of NOKSI with rhANGPTL4 full-length (A4 FL) and NRP1 inhibitor (EG00229) 10µM and 100µM

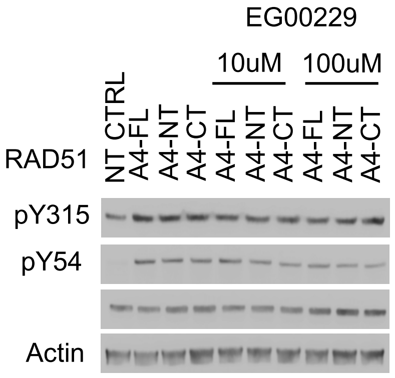

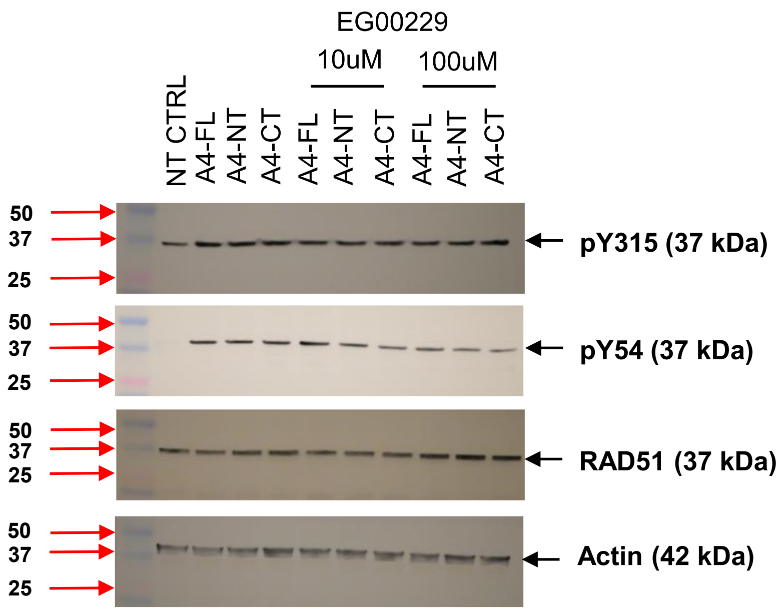

Figure 5I: Cisplatin and Imatinib (ABL1 inhibitor) in CAL27 cells

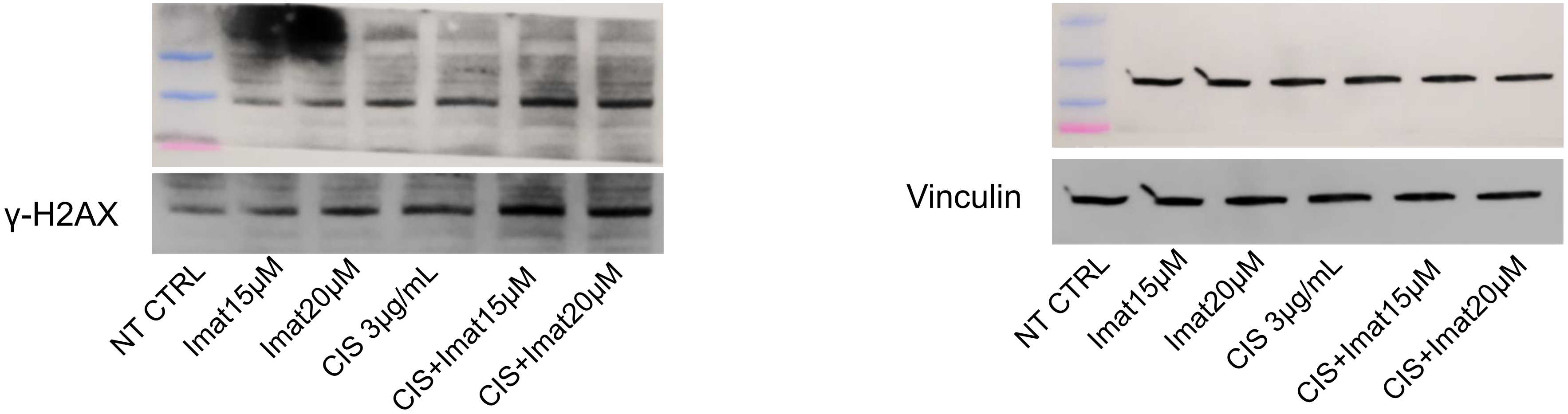

Figure 5K: Cisplatin and Dasatinib (ABL1 inhibitor) in CAL27 cells

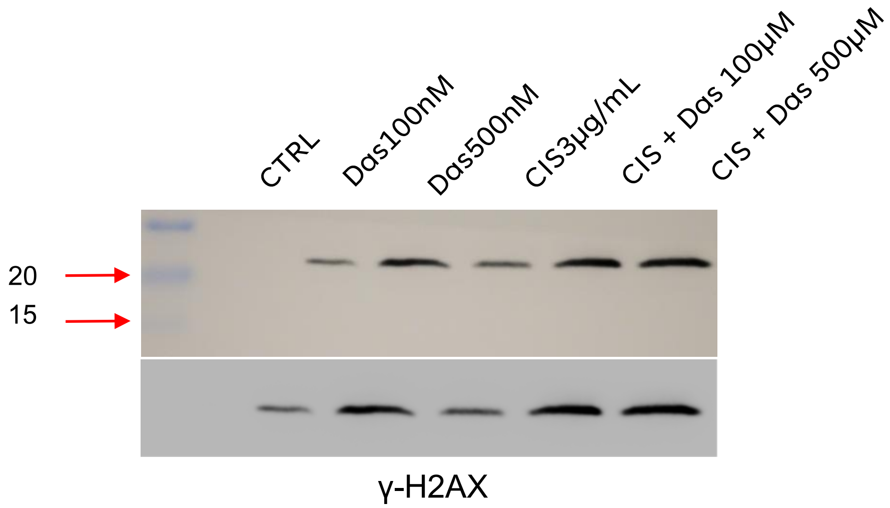

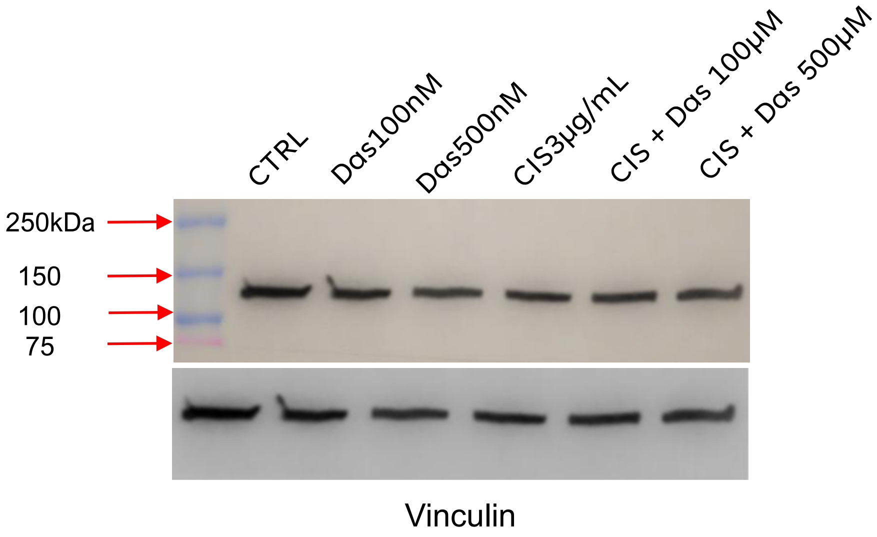

Figure 5M: Cisplatin and Dasatinib (ABL1 inhibitor) in HN13 cells

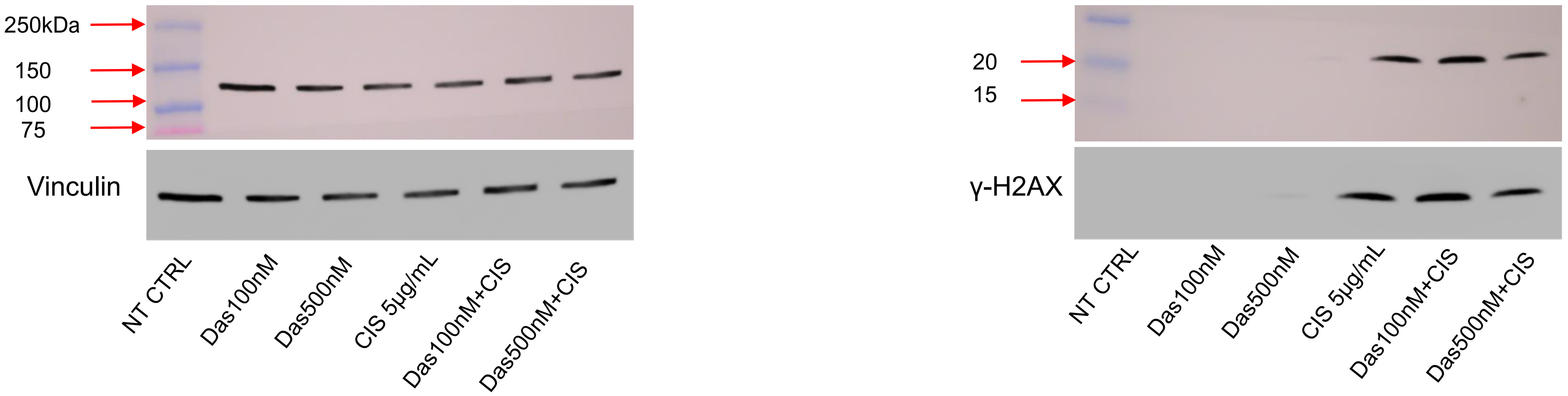

Figure 5O,P: Western blot analysis of RAD51 Y315 and Y54 phosphorylation upon treatment of NOKSI with rhANGPTL4 full-length (A4 FL) and knockdown of ABL1

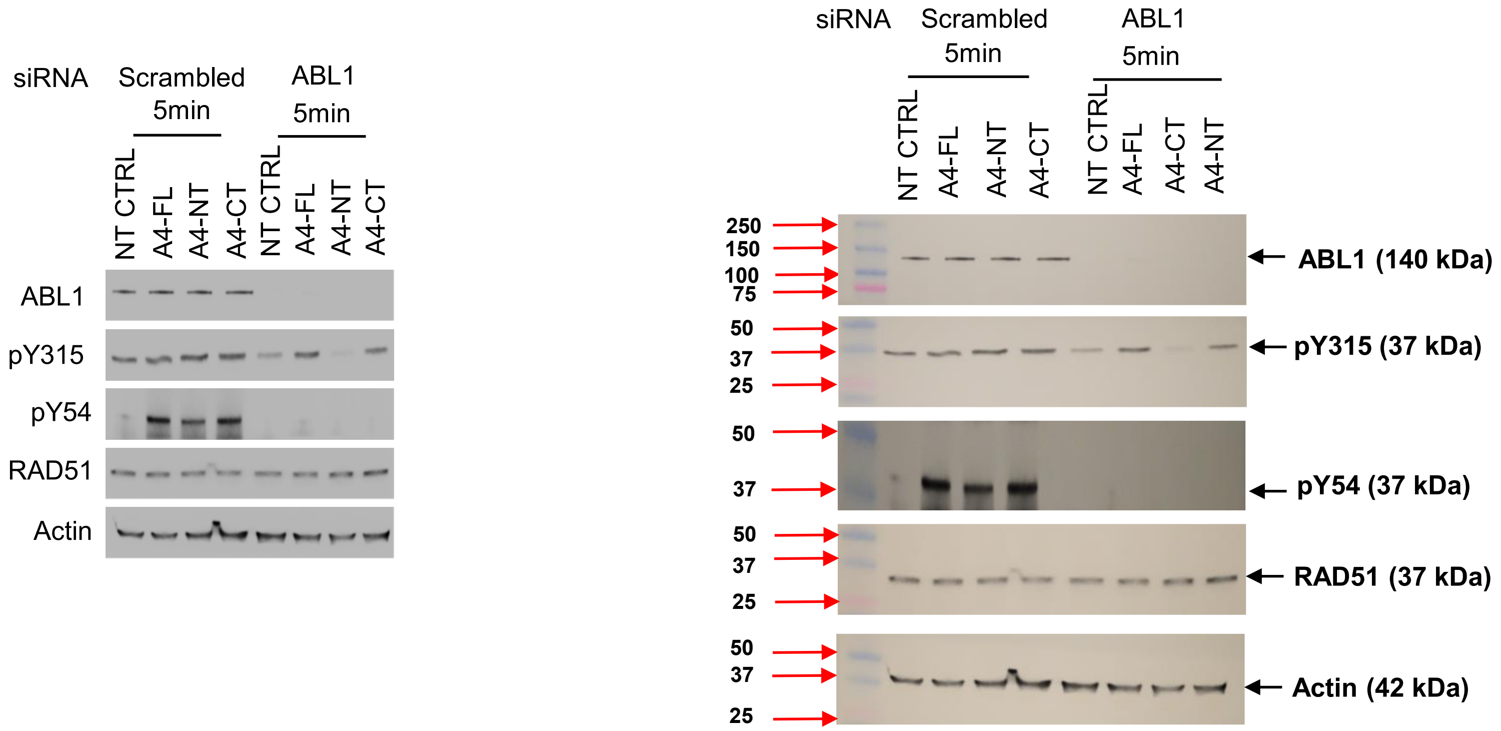

Supplementary Figure 1A: RAD51 Expression following HN4 and HN4 A4 OE treatment with Cisplatin

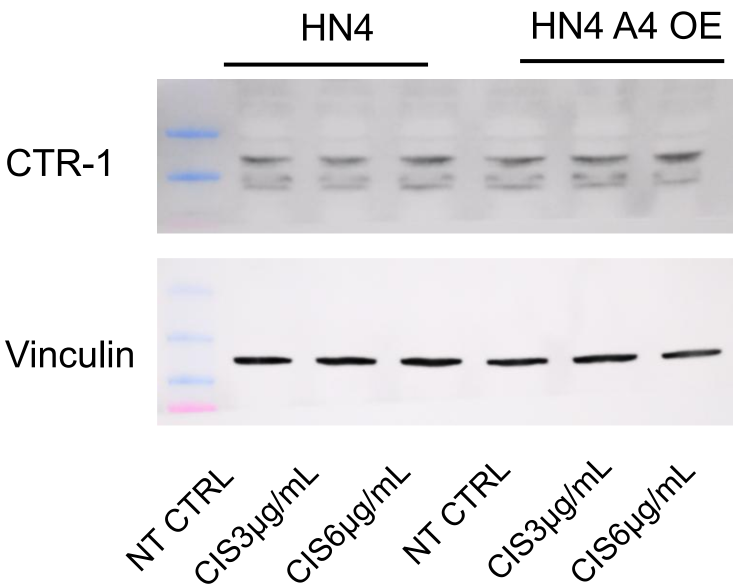

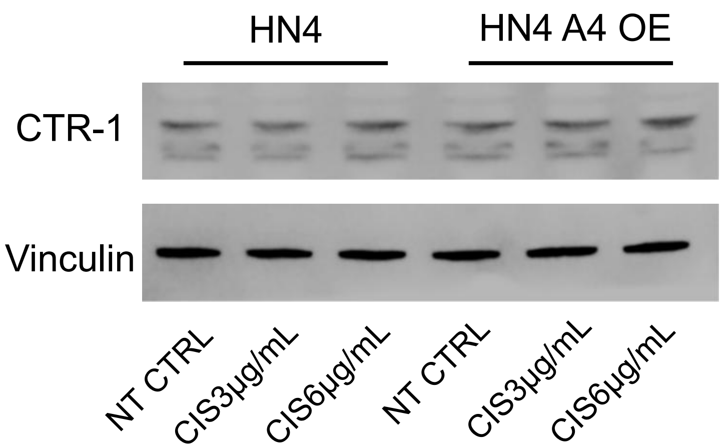

Supplementary Figure 2A: ANGPTL4 loss increases DNA damage in HNSCC cells in response to cisplatin

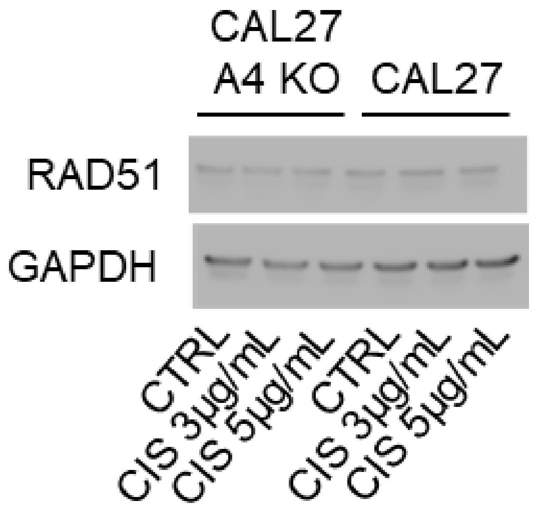

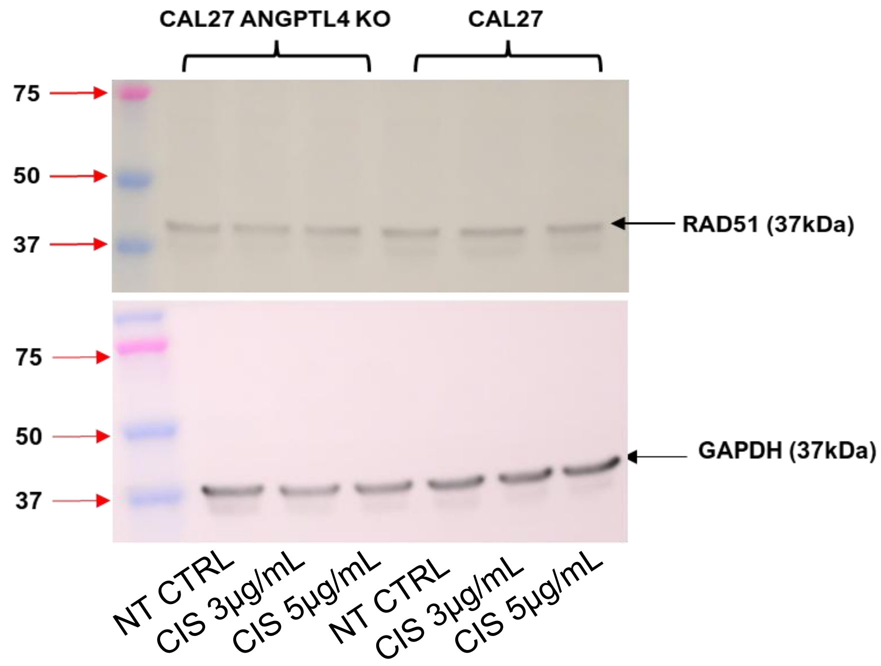

Supplementary Figure 3E: NRP-1 Inhibition (EG00229) impact on DNA damage response in HN13 cells

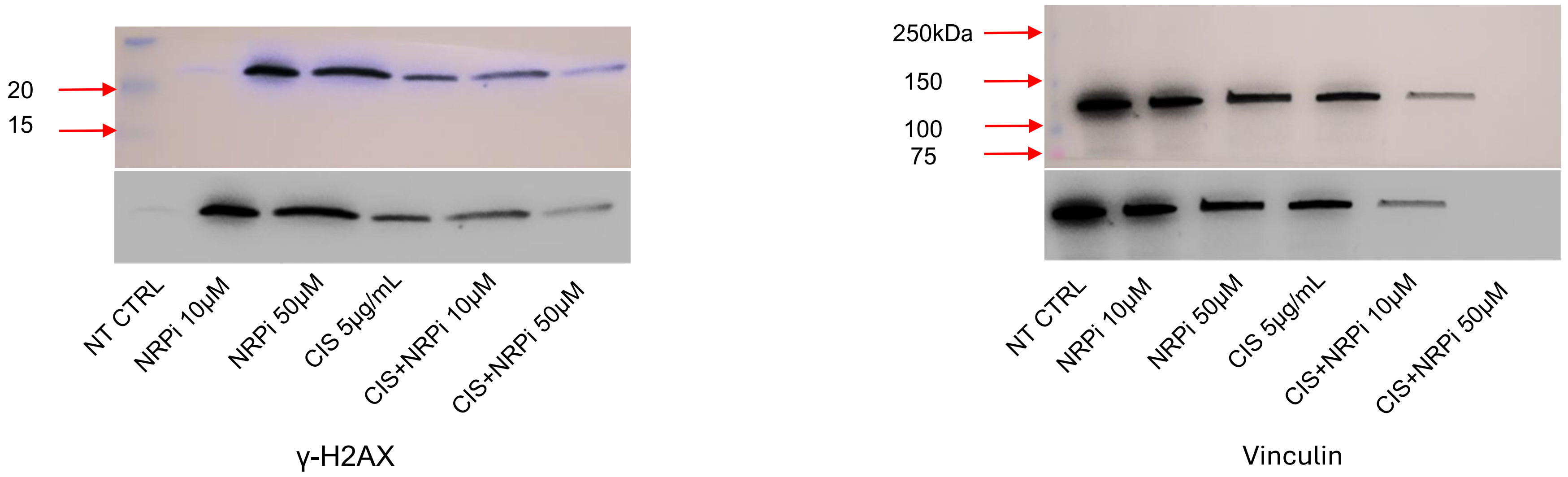

Supplement: Supplementary file 3 — Dataset S02 (PDF) [file pnas.2510265123.sd02.pdf]
